# Early biofouling colonization stages: Implications for operation and maintenance planning in marine renewable energy projects

**DOI:** 10.12688/openreseurope.14854.1

**Published:** 2022-09-05

**Authors:** Pedro Almeida Vinagre, Gonçalo Fonseca

**Affiliations:** 1 Environment and Licensing, WavEC Offshore Renewables, Lisbon, Portugal

**Keywords:** Biofouling, Colonization, Macrofouling, Marine Renewable Energy, Non-indigenous species, Operations and Maintenance

## Abstract

**Background:** Marine biofouling is a threat to industries working in the marine environment, representing enormous costs associated with equipment impairment and loss of performance. In the Marine Renewable Energy (MRE) and other maritime sectors which operate at sea for long periods, an important aspect of biofouling is related to the type and frequency of maintenance.

**Methods:** This study investigated important parameters of macrofouling (for example composition, including the presence of non-indigenous species, thickness, and weight) from communities growing on small-scale wave energy components in marine conditions. The trials were performed during short periods of submersion (one to eight weeks) in the seasons when the colonisation process should be most intensive (spring, summer, and autumn). Furthermore, the frictional resistance forces generated to scrape the biofouling from those artificial components were investigated.

**Results:** Overall, results show that while biofouling growth in early colonization stages might not present great detrimental effects to wave energy components, although marine corrosion and the settlement of non-indigenous species (NIS) should be factors of concern.

**Conclusions:** It is suggested to perform biofouling-related maintenance activities after the peak of maximum growth and reproduction (during the warmer seasons in temperate to cold environments) to reduce the number and frequency of activities. NIS can be detected very early in the colonization process, highlighting the importance of biofouling monitoring and the implementation of biosecurity risk assessment plans early in the operational stage of MRE projects.

## Introduction

Marine biofouling is a natural process which poses great challenges to the maritime sectors ( *e.g.* marine renewable energy, oil and gas, shipping, aquaculture), most often resulting in loss of structural integrity, performance and productivity representing enormous costs to the maritime sectors ( *e.g.* Bannister *et al.,* 2019; Loxton *et al.,* 2017; Satpathy *et al.,* 2010; Schultz *et al.,* 2011; Titah-Benbouzid & Benbouzid, 2017).

With regards to the marine renewable energy (MRE) sector (including ocean energy and offshore wind), biofouling (namely macrofouling) adds substantial weight to the equipment and structures, and increases their surface diameter and roughness, resulting in increased drag of moving parts and loss of equipment functionality and performance ( *e.g.* Blair *et al.,* 2014; Jusoh & Wolfram, 1996; Titah-Benbouzid & Benbouzid, 2017; Yang *et al.,* 2017). Moreover, biofouling may induce or accelerate corrosion in the equipment: larger organisms (macrofouling) facilitate microbiologically induced/influenced corrosion ( *e.g.* Jia *et al.,* 2019; Videla & Herrera, 2005) which is initiated by microbial communities (microfouling) growing under the macrofoulers in oxygen-depleted conditions; corrosion may further be accelerated by some macrofoulers via mechanical or chemical actions used to adhere to (acorn barnacles) or perforate (boring bivalves) substrates ( *e.g.* Blackwood *et al.,* 2017; Kleemann, 1996).

Another concern related to biofouling is that it creates opportunity for non-indigenous species (NIS) to settle and spread across geographical regions. This has been the case of several MRE structures and equipment deployed at sea in the last years ( *e.g.* Adams *et al.*, 2014; De Mesel *et al.*, 2015; Kerckhof *et al.,* 2011; Langhamer, 2012; Nall *et al.*, 2017).

To overcome the biofouling challenge to the maritime sectors, several anti-fouling (AF) solutions have been developed over the last decades. However, biofouling structure and growth varies greatly depending on the geographical location, season, depth, and substrate composition and roughness, among many other factors ( *e.g.* Hellio & Yebra, 2009; Vinagre *et al.*, 2020). Hence, to date, no AF solution is simultaneously applicable worldwide and efficient against all biofouling organisms. Furthermore, the AF industry may face a further challenge with the increase of seawater temperature and acidification associated with climate change ( Dobretsov *et al.,* 2019): First, the increased temperature and acidification have detrimental effects to many marine organisms, especially calcifying organisms such as barnacles, mussels, and tubeworms, which often make the bulk of biofouling and which are generally targeted by AF solutions; second, increased temperature and acidification may lead to changes in the durability and efficacy of some AF solutions ( Dobretsov, 2009; Dobretsov *et al.,* 2019). Hence, mechanical techniques ( *e.g.* cleaning, brushing, scraping) appears at present the only method capable of being totally efficient against biofouling worldwide, ensuring that no organism (or part of it, for example barnacle shells), remains on the equipment.

With regards to the MRE sector, monitoring of biofouling (namely macrofouling) often analyses composition, abundance (as biomass, density, or coverage) and/or thickness parameters after the equipment has been deployed in marine conditions for several continuous months or years. This allows the biofouling communities to become more complex, capturing high values of those biofouling parameters which could represent worst-case scenarios. On the other hand, understanding the structure of biofouling composition and its magnitude in early colonization stages, especially during different seasons, is of utmost importance. This allows, for example, to estimate minimum/maximum time intervals to perform maintenance tasks and to understand which could be the best periods to deploy equipment at sea. In terms of conservation, it allows to early detect the presence of NIS populations in the area and initiate mitigation measures to their proliferation.

The activities that lead to the present work were developed under the Horizon 2020 project WaveBoost which designed and developed an advanced power take off (PTO) system for enhanced reliability and performance of Wave Energy Converters and were encompassed in the work package dedicated to performance assessment and improvement. The wave energy technology tested under this project was developed by CorPower Ocean, where the energy converter is of the point absorber type, with a heaving buoy on the surface absorbing energy from ocean waves. The activities included the assessment of (i) biofouling composition (including the presence of NIS) and key biofouling parameters (thickness, richness, biomass, and density), and (ii) the frictional resistance forces generated during the scraping of biofouling, from small-scale samples of the rods of the CorPower Ocean’s PTO deployed in marine conditions.

The aim of this work was to increase understanding on biofouling community structure in early colonization stages (during short, increasing periods of one to eight weeks of submersion) across different seasons (spring, summer, and autumn) and, based on that, to delineate some recommendations on biofouling management which could aid the implementation and the planning of operations and maintenance activities of MRE projects.

## Methods

### Study site and sampling

**Figure 1.**
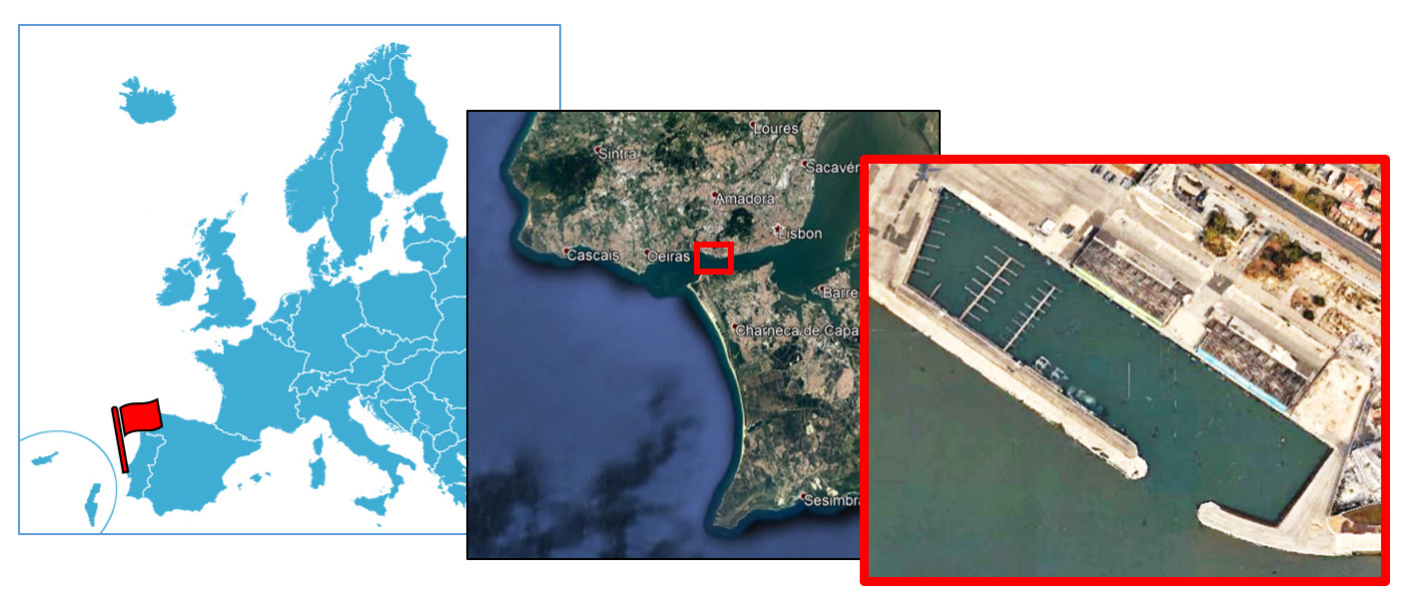
Test site location in south-west Europe.

The Pedrouços harbour (Lisbon, Portugal; 38º41’38’’N, 9º13’31’’W) is located in a temperate climate region on the south-western Atlantic coast of Europe, at about 6 km upstream the mouth of Tagus estuary in Lisbon, Portugal ( Figure 1). The harbour serves a restricted number of small fishing vessels. Openings in the harbour walls allow for seawater to pass through creating light wave action (maximum 0.5 m) and water circulation.

At the harbour, depth in the area of sampling ranges between ~5 m at low tide and ~8 m at hight tide.

**Figure 2.**
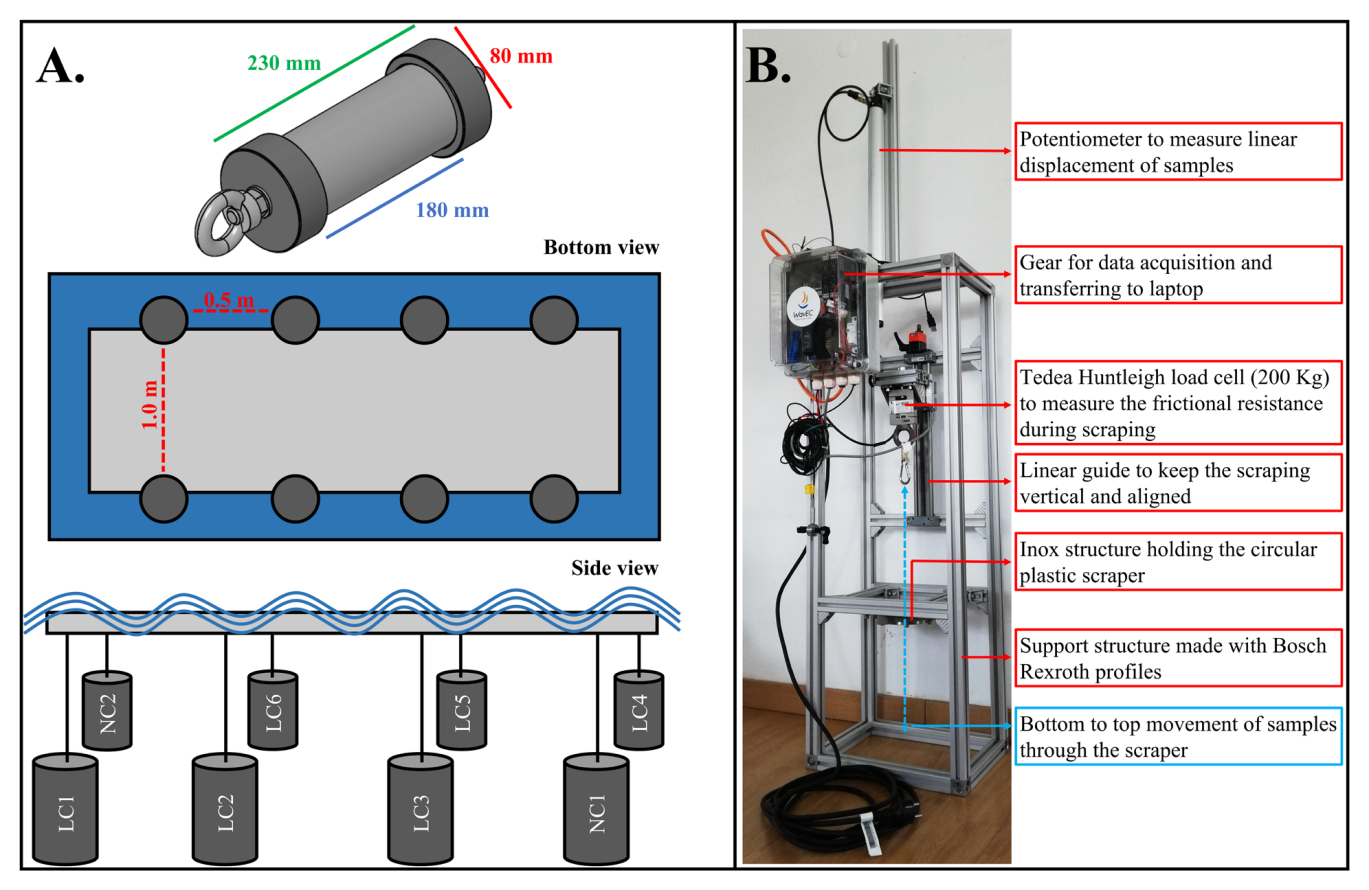
**A**. Cylinders and deployment design. **B**. The setup used for cylinders scraping.

Eight cylindrical samples (230 mm × 80 mm; colonizable area: 180 mm × 80 mm) representing rods of a hydraulic PTO system were placed (suspended in a floating rig) submerged at ~3 m depth for different periods of time (one, two, three, four, five, six and eight weeks, henceforth designated as 1–8W) between May and November of 2019 ( Table 1; Figure 2A.).

**Table 1.** Biofouling and frictional resistance sampling events. Each grey box corresponds to a continuous submersion period of samples (numbers identify the number of submersion weeks). Light grey corresponds to frictional resistance data available for the analyses.

Month	Spring								Summer										Autumn							
Sample	May				June				July		Aug				Sep				Oct				Nov			
LC1				1		2				3		1				1		4								
LC2				2						4						1		4								
LC3				4						4						2			6							
LC4				4						5						2			6							
LC5				4						5						3			8							
LC6				4						5						3			8							
NC1				4						4		4														
NC2				4						5																

The cylinders were made of S355 steel and were coated with two different anti-corrosion treatments (for industry/research-based reasons):

- Six out of the eight samples were coated with a laser cladded alloy (similar to Stellite) based on corrosion-resistant metals (stainless steel, nickel, chrome, and cobalt; kept confidential to protect commercial interests); these six samples are hereafter named LC1, LC2, LC3, LC4, LC5 and LC6.
- Two out of the eight samples were coated with electroplated nickel-chromium; these two samples are hereafter named NC1 and NC2.

The NC1 and NC2 cylinders showed signs of corrosion during trials in September 2019 and were not used for further trials (data of biofouling growing in those conditions were discarded from the analyses to avoid biased results).

The cylinders were processed following a stepwise methodology (example is given for sample LC1):

1. Each sample was retrieved from field after a first submersion period (shown in Table 1; in the case of LC1 it was after one week of submersion in May);
2. In the laboratory, each sample thickness (mm) was measured using a watertight digital calliper;
3. After, each sample was placed in the test rig conceived by WavEC and CorPower Ocean ( Figure 2B.) submerged in water and was scraped with a circular plastic scraper. The aim was to recover the biofouling from the cylinders and to measure the frictional resistance forces created during the removal of biofouling. The frictional resistance data was acquired by force and displacement measurements using a loadcell and a potentiometer, respectively. These sensors were connected to the cylinder shaft that was pulled along a motorized linear guide;
4. After being scraped from the cylinders, the biofouling was sieved gently through a 0.5 mm mesh sieve and the organisms retained were processed. Each cylinder sample was then gently cleaned using a sponge and liquid detergent and was re-deployed in the field for another submersion period (in the case of LC1, for submersion during the second and third weeks of June 2019). The plastic scraper was replaced by a new one to avoid any indentations which could scratch the next sample;
5. Upon processing, all biofouling organisms were identified, counted, and weighed. Taxonomy for macroinvertebrates and macroalgae was done to the lowest taxonomic level possible and was standardized in accordance to the World Register of Marine Species ( WoRMS) and the AlgaeBase, respectively.

In parallel to biofouling sampling, seawater temperature (°C), salinity, dissolved oxygen (DO; mg L ^-1^) and total chlorophyll (Chl.; µg L ^-1^) were measured at 3 m depth using a YSI ProDSS handheld multiparameter probe. With no particular reason, a greater number of measurements coincided with low tides (spring: two out of two sampling events; summer: three out of six sampling events; autumn: three out of five sampling events).

### Data analysis

All statistical analyses were performed with PRIMER 6 + PERMANOVA software ( Anderson *et al.,* 2008; Clarke & Gorley, 2006). The PERMANOVA, SIMPER, and PCO analyses can be performed using open-source software such as R (using the Vegan package in R) or PAST (except SIMPER; PAST available from the University of Oslo Natural History Museum website). A PRIMER trial version can be downloaded from the PRIMER website.

***Seawater parameters.*** For each seawater parameters (temperature, salinity, DO, and Chl.), statistically significant differences among seasons were tested using permutational multivariate analysis of variance (PERMANOVA). The design included one fixed factor, ‘Season’ (three levels: spring, summer, and autumn). The Euclidean distance was used in the calculation of the resemblance matrix. The statistical significance of variance components was tested using 999 permutations and unrestricted permutation of raw data, with a significance level of α = 0.05.

***Biofouling parameters.*** Prior to data analysis, macroinvertebrate density was standardized to number of individuals per square metre (ind m ^-2^), and invertebrate and algae biomass were standardized to grams of fresh weight per square metre (g FW m ^-2^).

Six biofouling parameters were used to describe the biofouling communities. Four were univariate parameters: number of taxa ( *Richness*), total biofouling biomass ( *TBiom*), total biofouling density ( *TDens*) and *Thickness*, and two were multivariate parameters: organisms biomass ( *BIOM*) and density ( *DENS*).

For statistical analysis of biofouling data, the feasibility of using the data of both cylinder treatments – LC and NC – together in subsequent analyses was first assessed. Statistically significant differences between the two treatments were tested using PERMANOVA applied individually to *Richness*, *TBiom*, *TDens, Thickness, BIOM*, and *DENS*. The statistical design included the fixed factors ‘Treatment’ (two levels: LC and NC), ‘Season’ (three levels: spring, summer, and autumn) and ‘Submersion’ (seven levels: 1, 2, 3, 4, 5, 6 and 8W) nested in ‘Season’. The Euclidean distance (univariate data) or Bray Curtis similarity (multivariate data) were used in the calculation of resemblance matrices, with addition of a dummy variable of the lowest value in the source data matrix. Prior to calculating the resemblance matrices, *TBiom, TDens, BIOM* and *DENS* data were square root-transformed. The statistical significance of variance components was tested using 999 permutations, with unrestricted permutation of raw data (univariate data) or permutation of residuals under a reduced model (multivariate data), with a significance level of α = 0.05. When the possible permutations were <100 the Monte Carlo *p* value was selected.

After, using the LC and NC data combined (because no statistical differences were previously found; see *Extended data*), statistical differences among seasons and among submersion periods within season were assessed individually for *Richness*, *TBiom*, *TDens* and *Thickness*. The statistical design included the factors ‘Season’ and ‘Submersion’ nested in ‘Season’, and the same options were used as for the previous PERMANOVA.

Following this, analysis of similarity percentages (SIMPER) was applied individually to *BIOM* and *DENS* to identify the taxa which contributed mostly to the statistical differences. First, dissimilarities among seasons were assessed using two-way crossed designs with factors ‘Season’ and ‘Submersion’. Then, dissimilarities among submersion periods within season were assessed selecting each season data and using a one-way design with the factor ‘Submersion’. For all SIMPER analyses a 95% cut-off was used, without transformation of data.

***Relation between the biofouling and seawater parameters.*** To visualize the seasonal relation between biofouling parameters ( *Richness, TBiom, TDens,* and *Thickness*) and seawater parameters (temperature, salinity, DO, and Chl.) a principal coordinates analysis (PCO) was conducted. To do this, the seawater parameters data were averaged per season and that value was used for each biofouling sample in that season ( *e.g.* spring water temperature was the same for the spring biofouling 1W, 2W, and 4W samples).

***Frictional resistance forces data.*** The friction forces data obtained by scraping biofouling from the sample cylinders were pre-processed to remove outliers, *e.g.* associated with the acceleration at start or deceleration at stop of the scraping system owed to the tightness of the plastic scraper to the samples.

## Results

**Figure 3.**
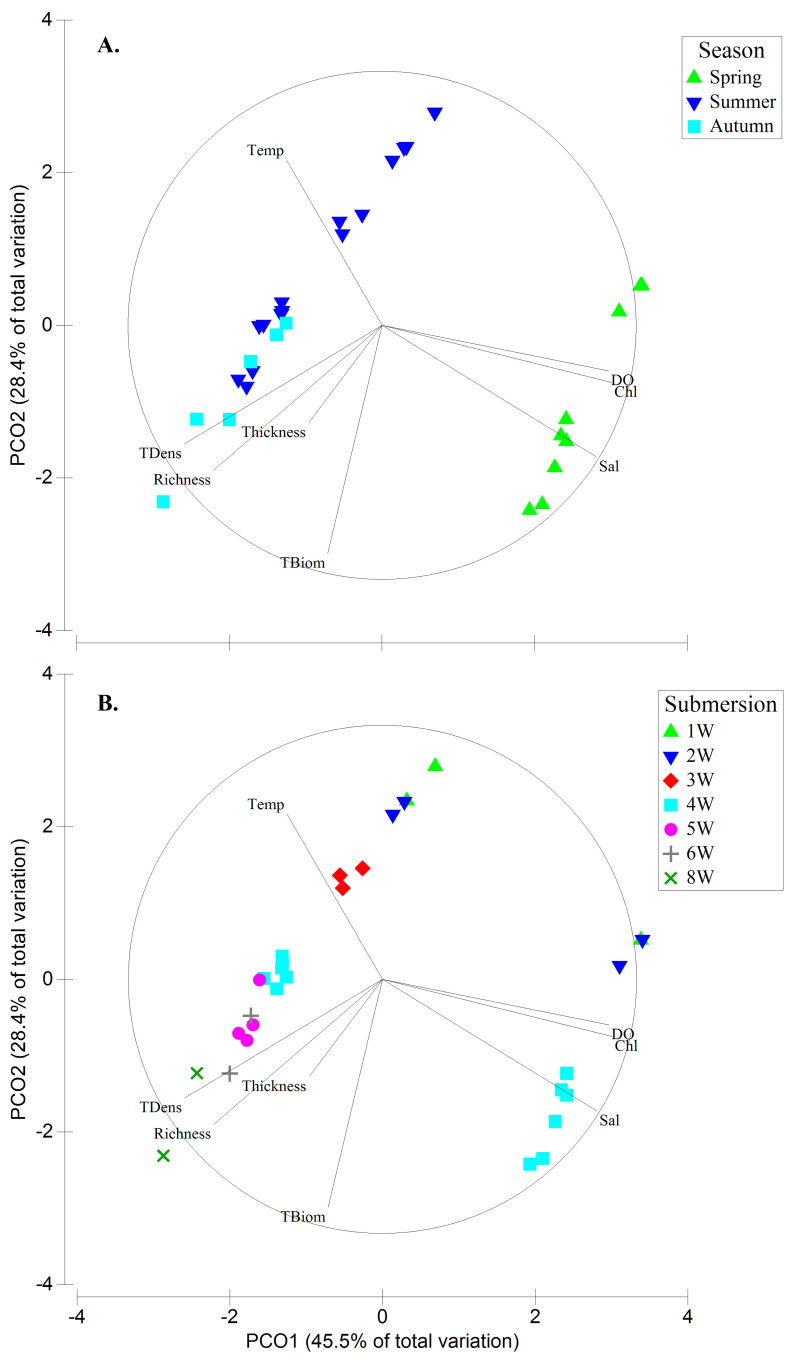
Principal Coordinates analysis (PCO) plot showing trends of seawater parameters (temperature, salinity, dissolved oxygen [DO] and total chlorophyll [Chl.]) and biological parameters ( *Richness, Total biomass [TBiom], Total density [TDens]* and *Thickness*) among seasons ( **A**.) and submersion periods ( **B**.).

Mean water temperature was greater in the summer, while spring registered greater salinity, DO, and Chl. (the latter two decreasing from spring to autumn) ( Table 2A., Figure 3). Statistically significant differences were found among seasons for water temperature, salinity, and Chl. ( *Extended data*).

**Table 2.** Seasonal values (mean ± standard deviation, except for Richness) for the seawater parameters (A.) and the biofouling parameters (B.). Greater numbers are presented in bold.

**A.**		Temperature (ºC)	Salinity	Dissolved Oxygen (mg L ^-1^)	Chlorophyll (µg L ^-1^)
Spring		16.8 ± 0.2	**40.6 ± 0.1**	**7.23 ± 0.29**	**2.63 ± 0.73**
Summer		**18.5 ± 1.2**	38.6 ± 1.1	6.83 ± 0.68	1.50 ± 1.13
Autumn		16.7 ± 1.6	38.9 ± 1.2	6.84 ± 0.21	0.76 ± 0.28
**B.**		*Richness*	*Thickness* * (mm)	*TBiom* * (g FW m ^-2^)	*TDens* * (ind m ^-2^)
Spring	1W	2	0.20 ± 0.00	0.03 ± 0.00	15.3 ± 0.0
	2W	6	0.25 ± 0.21	0.71 ± 0.40	38.3 ± 54.1
	4W	**15**	**1.05 ± 0.22**	**35.6 ± 10.6**	**512.6 ± 180.7**
Summer	1W	4	0.11 ± 0.18	0.09 ± 0.16	12.1 ± 20.9
	2W	9	0.10 ± 0.00	0.58 ± 0.14	63.3 ± 12.8
	3W	16	0.76 ± 0.55	5.7 ± 3.3	482.3 ± 297.4
	4W	**19**	1.65 ± 0.66	13.1± 2.5	2224.6 ± 478.0
	5W	**19**	**1.90 ± 0.26**	**23.8 ± 8.8**	**2694.8 ± 373.6**
Autumn	4W	15	**0.49 ± 0.01**	21.6 ± 0.31	1039.9 ± 217.4
	6W	14	0.44 ± 0.47	35.6 ± 15.1	2929.9 ± 511.5
	8W	**18**	0.37 ± 0.37	**44.4 ± 12.6**	**4928.4 ± 217.4**

* Mean ± standard deviation

**Figure 4.**
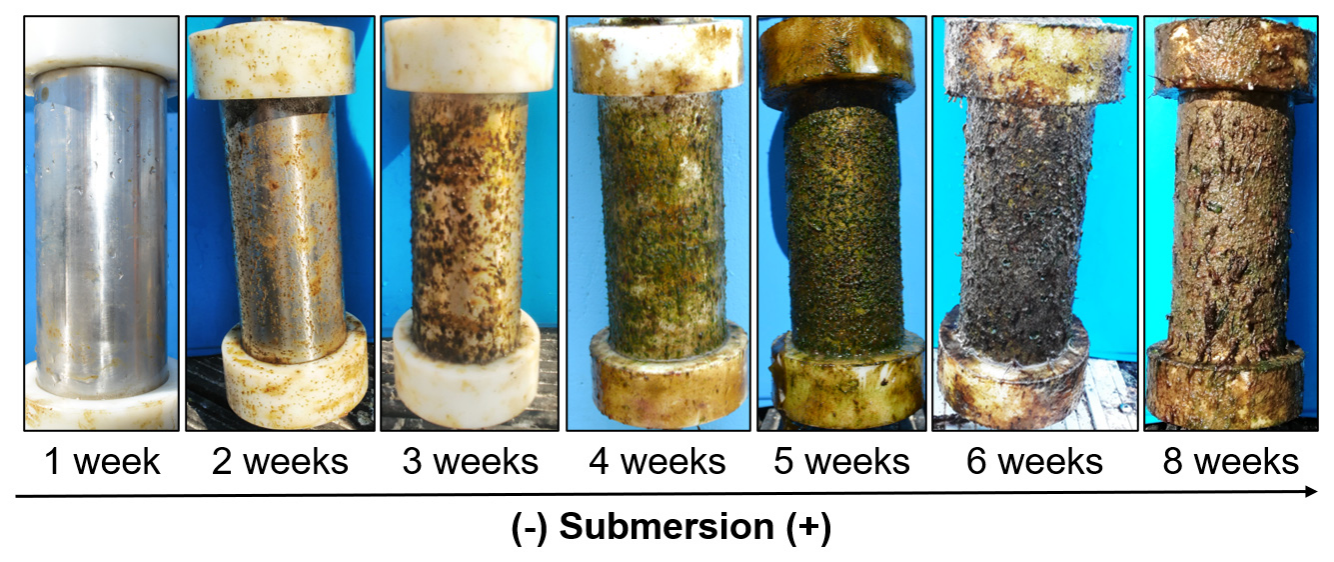
Biofouling growth after one, two, three, four, five, six and eight weeks of samples submersion.

The biofouling growth was noticeable with increasing submersion time of samples ( Figure 3, Figure 4, Table 3). *Richness* (statistically different among all seasons; *Extended data*) and *Thickness* (statistically different between summer and the other seasons; *Extended data*) were greater in summer at the maximum submersion of 5W. *TBiom* (statistically different between autumn and the other seasons; *Extended data*) and *TDens* (statistically different among all seasons; *Extended data*) were greater in autumn at the maximum submersion of 8W ( Table 2B.).

**Table 3.** List of taxa found in this study, showing their presence (in grey) across submersion periods (1–8W) within each season. Total macroalgal and macroinvertebrate taxa are presented per submersion period and per season at the bottom. The number of occurrences (occ.) of each taxon in the study is shown on the right side. Greater numbers are presented in bold.

Group			Taxa	Spring			Summer					Autum			Occ.
				1W	2W	4W	1W	2W	3W	4W	5W	4W	6W	8W	
**Macroalgae**	Ph. Chlorophyta	Or. Ulvales	*Ulva* sp. (tubular-like form)												**10**
			*Ulva* sp. (leaf-like form)												**10**
	Ph. Rhodophyta	Or. Ceramiales	cf. *Tiffaniella capitata*												7
			cf. *Pterothamnion crispum*												5
			*Polysiphonia* sp.												8
			cf. *Halurus flosculosus* / *Bornetia secundiflora*												7
		Ph. Rhodophya	Rhodophyta N.I.												8
	Cl. Phaeophyceae	Or. Ectocarpales / Or. Sphacelariales	*Hincksia* sp. / *Sphacelaria* sp.												**9**
**Macroalgal taxa per submersion period**				1	4	**9**	1	7	8	**9**	**9**	**9**	7	**9**	
**Macroalgal taxa per season**				**9**			**9**					**9**			
**Macroinvertebrates**	Ph. Bryozoa	Ph. Bryozoa	Bryozoa N.I.												8
	S.Ph. Crustacea	Or. Amphipoda	Amphipoda N.I.												**9**
			*Caprella equilibra*												7
		Or. Decapoda	cf. Anomura N.I. / Brachyura N.I.												3
			cf. *Pasiphaea sivado*												5
		Or. Isopoda	Gnathiidae N.I.												2
			*Tanais dulongii*												7
		Or. Sessilia	Barnacles ( *Perforatus perforatus*, *Austrominius modestus*)												**10**
	Cl. Pycnogonida	Or. Pantopoda	*Ammothella longipes*												1
	Cl. Polychaeta	F. Serpulidae	*Spirobranchus* sp.												7
		F. Syllidae	Syllidae N.I.												2
	Ph. Mollusca	Cl. Bivalvia	*Mytillus galloprovincialis*												2
		Cl. Gastropoda	cf. *Crisilla semistriata*												1
**Macroinvertebrate taxa per submersion period**				1	2	**6**	3	2	8	**10**	**10**	6	7	**9**	
**Macroinvertebrate taxa per season**				6			**11**					9			
**Total taxa per submersion period**				2	6	**15**	4	9	16	**19**	**19**	15	14	**18**	
**Total taxa per season**				15			**19**					18			

The above trends were reflected by some species succession in the colonization process ( Table 3). For example, after one week of submersion, only the opportunistic green algae ( *Ulva* sp.), barnacles ( *Perforatus perforatus* and the NIS *Austrominius modestus*) and bryozoans were recorded; after two weeks, filamentous brown algae ( *Hincksia* sp./ *Sphacelaria* sp.), red algae ( *e.g.* from the order Ceramiales) and several crustaceans fauna amphipods were observed; after three or more weeks, several other macroalgal and macroinvertebrate taxa joined the biofouling communities.

The SIMPER analyses using the organisms’ individual biomass ( *BIOM*) and density ( *DENS*) were largely in agreement with the overall trends in total biomass ( *TBiom*) and total density ( *TDens*). Using *BIOM*, nine taxa were the main contributors (cut-off 95%) for the dissimilarities among seasons and among submersion periods within season ( *Extended data*). With few exceptions, the biomass of those taxa was greater in autumn, and in every season increased with increasing submersion period ( *Extended data*). Using *DENS*, seven taxa were the main contributors to the dissimilarities ( *Extended data*). Although most of these organisms, and especially barnacles, registered greater density in the summer, the greatest values were presented by Amphipoda in autumn. With few exceptions, those taxa showed increasing density with increasing submersion period within each season ( *Extended data*).

**Figure 5.**
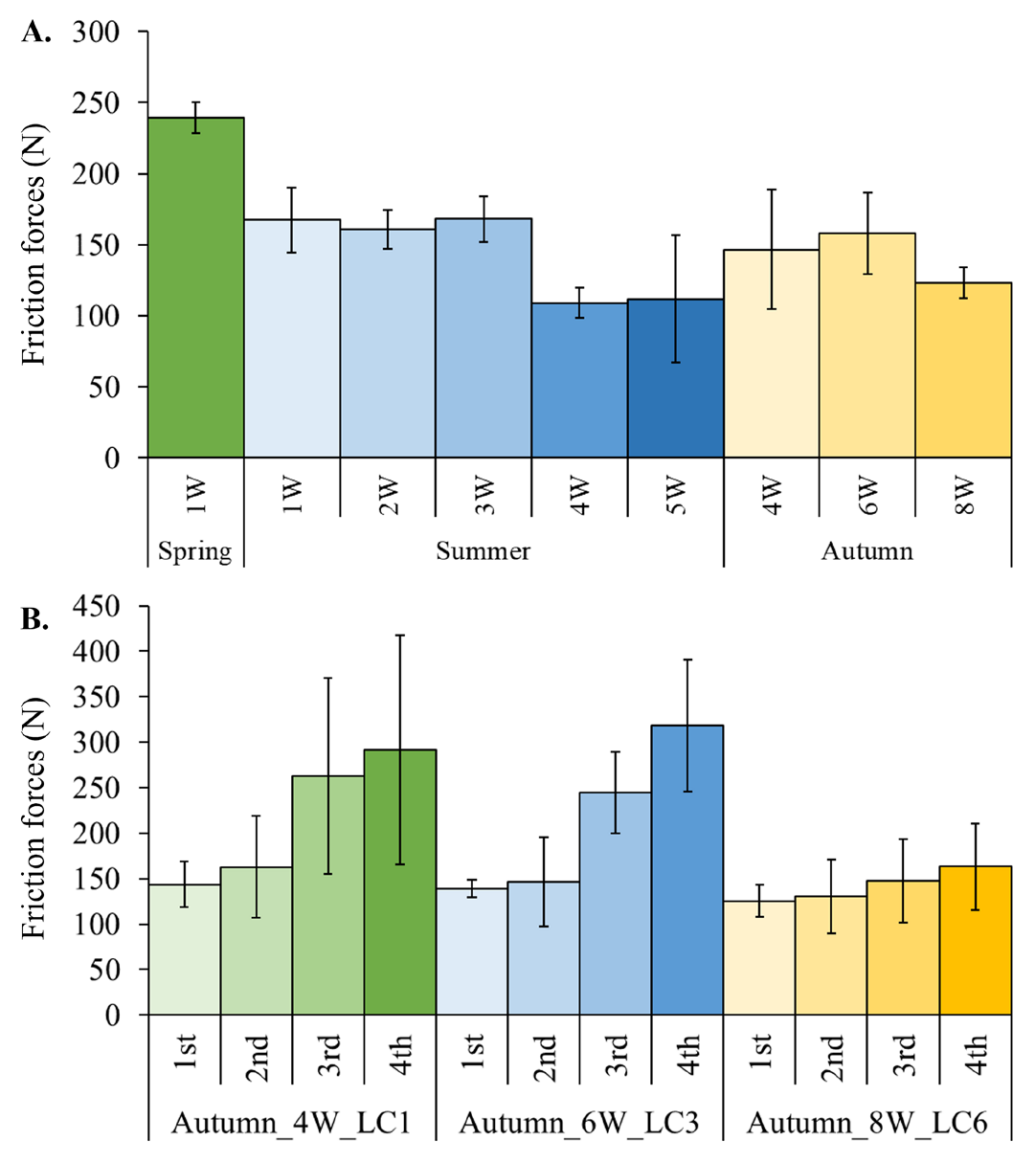
**A**. Frictional resistance forces (mean ± standard deviation) across submersion periods (1-8W) in different seasons. **B**. Frictional resistance forces (mean ± standard deviation) from four subsequent scrapings of three different samples.

With regards to the frictional resistance forces created when scraping the biofouling from the cylindrical samples, the trend was slightly contrary to that of the biofouling growth. Mean friction forces were about 250 N at 1W in spring and appeared to decrease to about 100 N with increasing submersion period until the 4W-5W submersion periods ( Figure 5A.). Also, mean friction forces increased with subsequent scrapings of the same sample ( Figure 5B.).

## Discussion

In this study, and as expected for this region, seasonal patterns were observed in the seawater parameters and the biofouling composition, richness, and abundance virtually accompanied seasonal changes. It was also expected that the greatest biofouling weight and density would be registered in spring and/or summer when the higher temperatures would favour the reproductive and growth rates of organisms ( *e.g.* Gili & Petraitis, 2009; Newell & Branch, 1980). Although both parameters were highest in autumn (at eight weeks of submersion), when considering the longest submersion period (four weeks) common among the three seasons, spring registered the greatest biomass (greatly associated with filamentous green and brown algae), while summer registered the greatest density (greatly associated with barnacles and amphipods). Thus, it could be expected that biofouling growth would be greater in these seasons instead of autumn if it was allowed for over four-five weeks. Accordingly, it is recommended that biofouling-related maintenance activities in temperate to cold regions are performed after warm seasons (for example summer) to avoid the elevated biofouling growth and, thus, to minimize the number of maintenance activities until the next season (for example the next spring) most suitable for the breeding, spawning, and settlement of numerous biofoulers ( *e.g.* Anil *et al.,* 2012; Hellio & Yebra, 2009; Kupriyanova *et al.,* 2001).

Biofouling biomass and thickness, both associated with macrofouling, are key biofouling parameters affecting several industries working in the marine environment ( *e.g.* Jusoh & Wolfram, 1996; Miller & Macleod, 2016; Tiron *et al.,* 2012; Titah-Benbouzid & Benbouzid, 2017; Yang *et al.,* 2017). In the present study, the values observed for those two parameters were quite below the values registered in more hydrodynamic areas ( *e.g.* nearshore/offshore) and longer submersion periods ( *e.g.* Vinagre *et al.,* 2022), even at a nearby harbour (OCEANIC project European Biofouling Database, Vinagre *et al.,* 2020). Accordingly, the weight and size of biofouling organisms (algae, bryozoans, barnacles, mussels, and calcareous tubeworms) which usually cause greater direct physical damage to structures/components (e.g. damaging the substrates or their protective coatings by boring into them, or when pulled by currents and waves) were also low. Therefore, it seems unlikely that biofouling growing for six to eight weeks under the present conditions would greatly increase the loading, frictional resistance, or surface diameter of structures/components. In fact, with regards to frictional resistance, it was found that during these early colonization stages the slippery nature of biofouling could be acting as a ‘lubricant’ leading to lower forces generated from scraping the samples in areas with biofouling compared to areas without biofouling. If the eight weeks can be accepted as a safe time interval to perform biofouling-related maintenance actions, then physical control, for example using water jetting/cavitation or acoustic methods ( *e.g.* Legg *et al.,* 2015), could be an option to maintain the components’ integrity and equipment functionality and performance. However, that will depend, among many other factors, on the type of structure/component and its functional requirements (for example, free-moving versus static), the location (for example, latitude, seawater temperature, distance to shore) and hydrodynamic conditions (for example, current velocity and wave exposure) of the site, the depth at which the structure/component is positioned (for example, surface versus mid water column), and season (for example, warm seasons versus cold seasons) ( *e.g.* Hellio & Yebra, 2009; Vinagre *et al.,* 2020).

Besides physical damage to structures/components by biofouling, detrimental issues may arise quickly concerning different types of corrosion ( *e.g.* Blackwood *et al.,* 2017; Jia *et al.,* 2019; Kleemann, 1996; Videla & Herrera, 2005). In the present study, corrosion was observed after one week of deployment in components untreated against marine-induced corrosion (stainless steel nuts used to tighten the caps) as well as in sections of NC samples (possibly owed to inefficient waterproofing of the untreated portion by the end caps) after four to five weeks of submersion in summer. This reinforces the importance of employing adequate anti-corrosion techniques to metallic substrates used in marine conditions (even if for short periods of time), for example by applying thermally sprayed aluminium which has proven capability to protect steel substrates ( *e.g.* Syrek-Gerstenkorn *et al.,* 2019; Syrek-Gerstenkorn *et al.,* 2020; Vinagre *et al.*, 2022) or laser-cladded materials ( *e.g.* Stellite) which in the present study showed good anti-corrosive efficiency, depending on the specific needs and cost.

Another concern, environment-related, is the settlement and propagation of NIS using the biofouling assemblages growing on MRE devices/structures. This is because NIS may pose serious ecological threats by competing with, predating on, and/or excluding indigenous organisms, affecting community composition and structure, and potentially causing habitat modifications ( *e.g.* Cook *et al.,* 2014; Crooks, 2002; Lengyel *et al.,* 2009), consequently affecting ecosystems functioning and ecosystem services provision.

In the present study, although some succession in biofouling colonization was observed, the presence of hard-fouling organisms such as barnacles after only one week of submersion is aligned with a more ‘probabilistic model’ of colonization ( Clare *et al.,* 1992; Maki & Mitchell, 2002) rather than a ‘successional model’, meaning that other organisms in the area, including NIS, can also settle in the artificial substrates early. At present, one NIS – the Australasian barnacle *A. modestus* – was found with great frequency and density. It is highly possible that its introduction in the area was caused by the shipping industry, considering the great traffic of ships (commercial, industrial and leisure) into and out of the Tagus Estuary. Unfortunately, after one to two weeks of samples submersion, some individual barnacle were very small and fragile and could not be well distinguished between *A. modestus* and *P. perforatus*. Hence, it is only certain that the NIS was registered after three weeks of submersion. As a vector of NIS propagation biofouling is comprised in legislative frameworks ( *e.g.* EU Directive 2008/56/EC, EU Regulation 1143/2014) that aim to prevent or manage the introduction and spread of NIS. Thus, it is valuable for the preservation of marine ecosystems and for MRE project developers to implement biosecurity risk management plans that can appropriately address biofouling and NIS propagation on their structures at sea ( *e.g.* Cook *et al.,* 2014; Payne *et al.,* 2014). This should be especially considered for MRE projects undertaken in areas where numerous NIS are registered, such as those next to shipping lanes, commercial harbours, or nearshore/offshore, for example in the North Sea ( *e.g.* De Mesel *et al.,* 2015; Kerckhof *et al.,* 2018; Vinagre *et al.,* 2020). An important outcome to the developers could be that such management plans support or complement environmental impact assessments, potentially increasing the acceptability of projects and speeding up the licensing process.

## Conclusions

The results of this study indicate that avoiding the greater availability of hard-fouling colonizers attaching to the devices early in the submersion period may contribute to reduce and delay further hard-fouling attachment with effects on materials preservation, increasing the extension of the period until the next cleaning operation. It is recommended to conduct biofouling-related maintenance activities after the peak of maximum growth and reproduction, generally occurring during the warmer seasons in temperate to cold environments. This way, the number of cleaning activities until the next growing season suitable for the breeding, spawning and settlement of numerous biofoulers can be reduced. The detection of NIS in this study after submerging artificial substrates for a short period (maximum of three weeks) highlights the importance of biofouling monitoring and the implementation of biosecurity risk assessment plans early in the operational phase of MRE projects as a good practice to maximise the prevention of NIS settlement.

## Data availability

### Underlying data

Zenodo: Early biofouling colonization stages: Implications for operation and maintenance planning in Marine Renewable Energy projects, https://doi.org/10.5281/zenodo.6974716 ( Vinagre & Fonseca, 2022a)

This project contains the following underlying data:

- Open Research Europe_Biological data.xlsx (Biofouling data)

Zenodo: Early biofouling colonization stages: Implications for operation and maintenance planning in Marine Renewable Energy projects, https://doi.org/10.5281/zenodo.6974740 ( Vinagre & Fonseca, 2022b)

This project contains the following underlying data:

- Open Research Europe_Friction forces_all samples.xlsx
- Open Research Europe_Friction forces_subsequential scrapings.xlsx

### Extended data

Zenodo: Early biofouling colonization stages: Implications for operation and maintenance planning in Marine Renewable Energy projects, https://doi.org/10.5281/zenodo.6962235 ( Vinagre & Fonseca, 2022c)

Data are available under the terms of the Creative Commons Attribution 4.0 International license (CC-BY 4.0) Underlying data.
