# Experimental insights on biofouling growth in marine renewable structures

**DOI:** 10.12688/openreseurope.14854.2

**Published:** 2024-06-19

**Authors:** Pedro Almeida Vinagre, Gonçalo Fonseca, Mário Vieira

**Affiliations:** 1 Environment and Licensing, WavEC Offshore Renewables, Lisbon, Portugal; 2 Engineering and Operations, WavEC Offshore Renewables, Lisbon, Portugal

**Keywords:** Biofouling, Colonization, Macrofouling, Marine Renewable Energy, Non-indigenous species, Operations and Maintenance

## Abstract

**Background:**

Marine biofouling is a threat to industries working in the marine environment, representing significant costs associated with equipment impairment and loss of performance. In the Marine Renewable Energy (MRE) and other maritime sectors which operate at sea for long periods, an important aspect of biofouling is related to the type and frequency of inspections and biofouling removal procedures.

**Methods:**

This study investigated important parameters of macrofouling ( *e.g.* composition, including the presence of non-indigenous species, thickness, and weight) from communities growing on samples that emulate tubular components of marine renewable devices. The trials were performed during short periods of submersion (one to eight weeks) in the seasons when the colonisation process should be most intensive (spring, summer, and autumn). Furthermore, the frictional resistance forces generated during the scraping of biofouling from those components were investigated.

**Results:**

Overall, results provide insights on the growth rates and removal requirements of biofouling in marine components. The results show that, while biofouling growth in early colonization stages might not present great detrimental effects to wave energy components, the consequent marine corrosion (fostered by biofouling) and the settlement of non-indigenous species (NIS) should be factors of concern.

**Conclusions:**

Performing biofouling-related maintenance activities after the peak of maximum growth and reproduction (during the warmer seasons in temperate to cold environments) is suggested to reduce the number and frequency of activities. NIS can be detected at very early stages in the colonization process, highlighting the importance of biofouling monitoring and the implementation of biosecurity risk assessment plans early in the operational stage of MRE projects.

## Introduction

Marine biofouling, i.e. the growth of microorganisms such as bacteria and microalgae (microfouling) and macroorganisms such as barnacles, mussels and macroalgae (macrofouling) on artificial substrates, is a natural process which poses great challenges to the maritime sectors ( *e.g.* marine renewable energy, oil and gas, shipping, aquaculture), most often resulting in loss of structural integrity, performance and productivity representing enormous costs to the maritime sectors ( *e.g.* Bannister *et al.,* 2019; Loxton *et al.,* 2017; Satpathy *et al.,* 2010; Schultz *et al.,* 2011; Titah-Benbouzid & Benbouzid, 2017).

With regards to the marine renewable energy (MRE) sector, biofouling (namely macrofouling) adds substantial weight to the equipment and structures (thus modifying their dynamic properties), and increases their surface diameter and roughness, resulting in increased drag of moving parts and loss of equipment functionality and performance ( *e.g.* Blair *et al.,* 2014; Jusoh & Wolfram, 1996; Titah-Benbouzid & Benbouzid, 2017; Yang *et al.,* 2017). Moreover, biofouling may induce or accelerate corrosion in the equipment: for example, larger organisms (macrofouling) facilitate microbiologically induced/influenced corrosion (MIC; *e.g.* Jia *et al.,* 2019; Videla & Herrera, 2005) which is initiated or exacerbated by microbial communities (microfouling) growing under the macrofoulers in oxygen-depleted conditions; corrosion may further be accelerated by some macrofoulers via mechanical or chemical actions used to adhere to (acorn barnacles) or perforate (boring bivalves) substrates ( *e.g.* Blackwood *et al.,* 2017; Kleemann, 1996).

Another concern related to biofouling of MRE structures (and others installed at sea) is that it creates opportunity for non-indigenous species (NIS) to settle and spread across geographical regions. This has been the case of several MRE structures and equipment deployed at sea in the last years ( *e.g.* Adams *et al.*, 2014; De Mesel *et al.*, 2015; Kerckhof *et al.,* 2011; Langhamer, 2012; Nall *et al.*, 2017).

To overcome the biofouling challenge to the maritime sectors, several anti-fouling (AF) solutions have been developed over the last decades, including mechanical removal systems, paints, and coatings, among others ( *e.g.* Hellio & Yebra, 2009; Vinagre *et al.*, 2020). However, biofouling structure and growth varies greatly depending on the geographical location, season, depth, and substrate composition and roughness, among many other factors. Hence, to date, no AF solution is simultaneously applicable worldwide and efficient against all biofouling organisms.

Biofouling management could become even more challenging in the near future due to climate change. First, ocean warming and acidification can contribute to changes in the expected biofouling communities’ structure and abundance ( Dobretsov *et al.,* 2019). For example, it could be detrimental to calcifying organisms ( *e.g.* barnacles, mussels, tubeworms) which often make the bulk of biofouling, and which might be replaced by soft macrofouling species such as ascidians. On the other hand, marine growth is generally more extensive and rapidly-developing in warmer regions, meaning that biofouling in temperate and polar seas could become more severe with ocean warming. Second, the increased temperature and acidification may lead to changes in the durability and efficacy of some AF solutions ( Dobretsov, 2009; Dobretsov *et al.,* 2019). Hence, at present, mechanical techniques such as biofouling removal, brushing, or scraping appear the most efficient against biofouling. Nonetheless, mechanical methods also present limitations. For example, their effectiveness is maximised on flat (or relatively flat) surfaces and is very much reduced on moving parts and surfaces with complex geometries. Furthermore, biofouling removal using mechanical techniques may damage or remove existing antifouling or anticorrosive coatings.

In the MRE sector, the monitoring of biofouling, namely macrofouling, often analyses composition, abundance (as biomass, density, or coverage) and/or thickness parameters after the equipment has been deployed in marine conditions for predetermined periods. Those generally extended periods (several continuous months) allow the biofouling communities to grow and become more complex, thus reaching great abundance and thickness, which for maintenance activities represent several constraints, for example covering the assets whose integrity and functionality need to be assessed ( *e.g.* Loxton *et al.*, 2017). On the other hand, understanding the structure and magnitude of biofouling in early colonization stages, especially during different seasons, is of utmost importance. This allows, for example, to estimate minimum/maximum time intervals to perform maintenance tasks and evaluate the best periods to deploy equipment at sea. It also allows to detect early the presence of NIS populations in the area and initiate mitigation measures to stop their proliferation.

**Figure 1.**
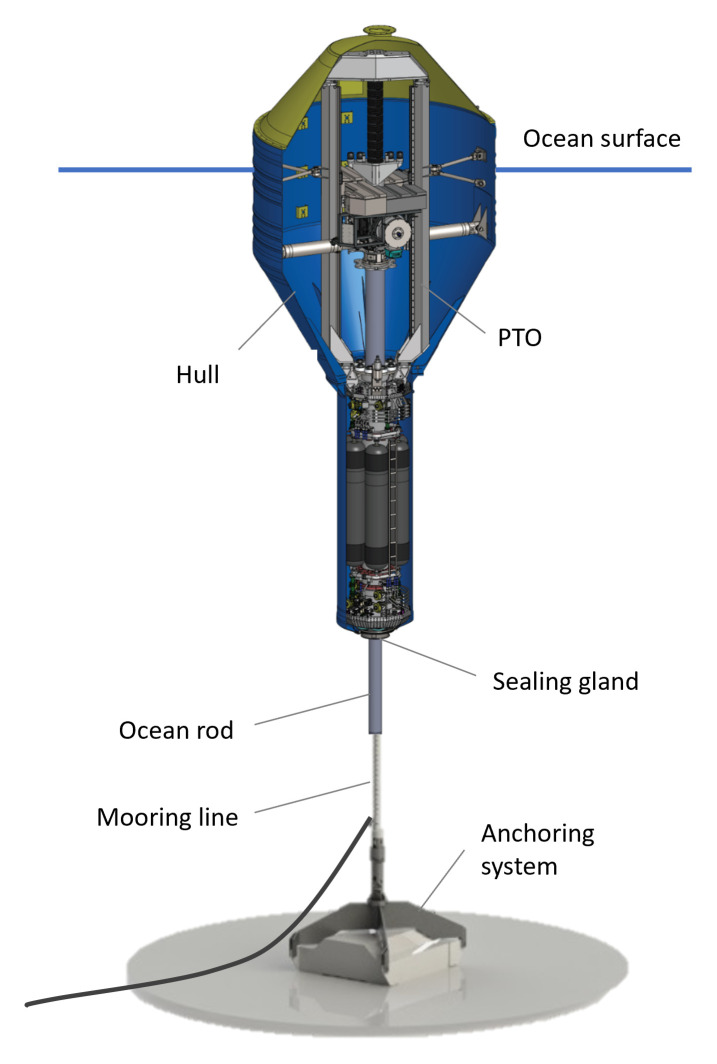
Schematic of CorPower Ocean wave energy device (source: CorPower Ocean).

The activities that led to the present work were developed under the Horizon 2020 project WaveBoost, which designed and developed an advanced power take-off (PTO) system for enhanced reliability and performance of Wave Energy Converters (WECs) and were encompassed in the work package dedicated to performance assessment and improvement. The WEC tested under this project was developed by CorPower Ocean. It is of the point absorber energy converter type with an oscillating part consisting of a heaving buoy which moves with the motion of incoming ocean waves and a stationary part consisting of the anchoring system, mooring line, ocean rod, and PTO system ( Figure 1). Particularly relevant for this research is the sealing system, the combination of the ocean rod surface and a seal gland, which acts as one of the interfaces between the oscillating and stationary parts. The sealing system is critical to the WEC’s functioning as it must allow low friction between the two parts to deliver maximum efficiency in the conversion of motion to electricity and prevent ingress of seawater inside the buoy hull ( Linden *et al*., 2022). Thus, CorPower Ocean devised a mechanical cleaning system (scraping system) to prevent biofouling growth which could damage the sealing system and compromise the WEC integrity and functioning.

The present work uses small-scale samples of CorPower Ocean’s device ocean rod with two objectives: (i) assess the biofouling growth, namely biofouling composition (including the presence of NIS), thickness, richness, biomass, and density, in the samples during short submersion periods, and (ii) assess the frictional resistance forces generated from scraping the biofouling from the samples using a prototype of CorPower Ocean mechanical cleaning system.

The overall aim is to increase understanding on biofouling community structure in early colonization stages (during short, increasing periods of one to eight weeks of submersion) across different seasons (spring, summer, and autumn) for coated steel-based tubular components of MRE devices and, based on that, to delineate some recommendations on biofouling management which could aid the implementation and the planning of operations and maintenance activities of MRE projects.

## Methods

### Study site and sampling

**Figure 2.**
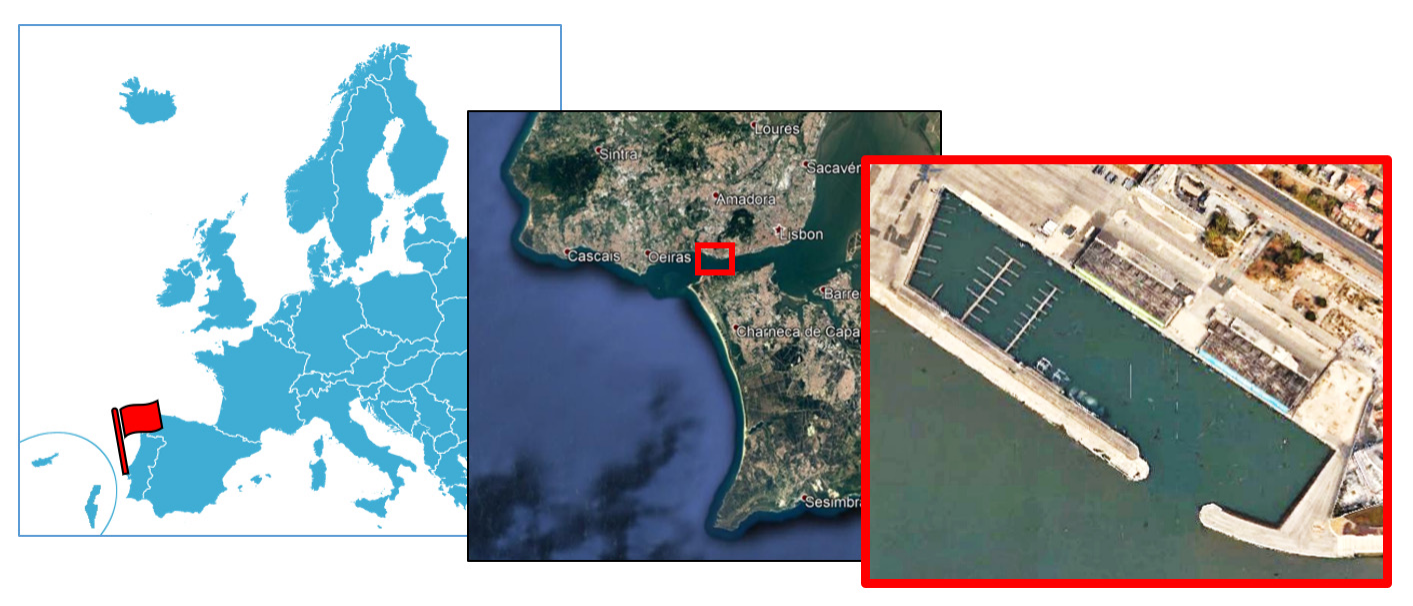
Test site location in south-west Europe.

The Pedrouços harbour (Lisbon, Portugal; 38º41’38’’N, 9º13’31’’W), where the experimental investigation of this research was produced, is located in a temperate climate region on the south-western Atlantic coast of Europe, at about 6 km upstream of the mouth of Tagus estuary in Lisbon, Portugal ( Figure 2). The harbour serves a restricted number of small fishing vessels. Openings in the harbour walls allow for seawater to pass through creating light wave action (maximum 0.5 m) and water circulation. At the harbour, depth in the area of sampling ranges between ~5 m at low tide and ~8 m at hight tide.

The study involved deploying and re-deploying eight cylindrical samples (230 mm × 80 mm; colonizable area: 180 mm × 80 mm) representing the rods of CorPower Ocean’s PTO system. The cylinders were made of S355 steel and were coated with two different anti-corrosion treatments (for industry/research-based reasons):

- Six out of the eight samples were coated with a laser-cladded alloy (similar to Stellite) based on corrosion-resistant metals (stainless steel, nickel, chrome, and cobalt; kept confidential to protect commercial interests); these six samples are hereafter named LC1, LC2, LC3, LC4, LC5 and LC6.
- Two out of the eight samples were coated with electroplated nickel-chromium; these two samples are hereafter named NC1 and NC2.

**Figure 3.**
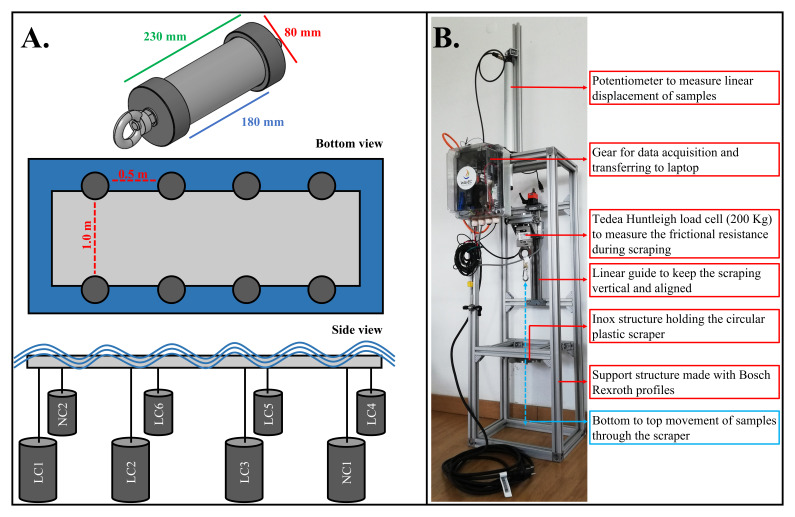
A. Cylinders and deployment design. **B**. The setup used for cylinders scraping.

The cylinders were suspended in a floating rig and submerged at ~3 m depth for different periods – one, two, three, four, five, six and eight weeks (henceforth designated as 1–8W) – between May and November of 2019 ( Table 1; Figure 3A.).

**Table 1.** Biofouling and frictional resistance sampling events. Each coloured box corresponds to a continuous submersion period of samples (numbers identify the number of submersion weeks). Light grey corresponds to samples without frictional resistance data available; Dark grey corresponds to samples scraped once; Black corresponds to samples that were scraped more than once.

Month	Spring								Summer										Autumn							
Sample	May				June				July		Aug				Sep				Oct			Nov				
LC1				1		2				3		1				1		4								
LC2				2						4						1		4								
LC3				4						4						2			6							
LC4				4						5						2			6							
LC5				4						5						3			8							
LC6				4						5						3			8							
NC1				4						4		4														
NC2				4						5																

The deployment and processing of the samples followed the stepwise methodology presented below, using LC1 as an example:

1) The cylinders were first deployed for a period; in the case of LC1, this cylinder was first deployed for 1W in May 2019;
2) Then, the cylinders were retrieved from the field for processing in the laboratory;
3) In the laboratory, biofouling thickness (mm) was measured as the highest point from the cylinder surface, associated with the presence of barnacles, bryozoans, or other organisms, using a watertight digital calliper;
4) After, each cylinder was placed in the test rig conceived by WavEC and CorPower Ocean ( Figure 3B.) submerged in water and biofouling was scraped with a circular plastic scraper: (i) The frictional resistance data were acquired at a 50 Hz frequency by force and displacement measurements using a load cell and a potentiometer, respectively. These sensors were connected to the cylinder shaft that was pulled along a motorized linear guide ( Figure 3B); (ii) Biofouling was kept for posterior processing. In some summer and autumn samples ( Table 1), subsequent scrapings were done until complete cleaning was achieved, to measure the frictional resistance forces generated from scraping decreasing levels of biofouling (macrofouling – biofilm – no biofouling);
5) After being scraped, the cylinders were gently cleaned using a sponge and liquid detergent. This allowed to totally clean the cylinder while avoiding abrasion and scratches. The plastic scraper was replaced by a new one to avoid any indentations which could scratch the next cylinder to be scraped;
6) The cylinders were then re-deployed in the field for another submersion period; in the case of LC1 it was for 2W in June 2019.
  It should be noted that the NC1 and NC2 cylinders showed signs of corrosion after re-deployments in September 2019, possibly owed to inefficient waterproofing of the untreated portion by the end caps. Corrosion can influence biofouling composition and growth, for example increasing the substrates roughness and physical-chemical properties. Therefore, data of biofouling growing in those samples in September 2019 were discarded from the analyses to avoid biased results and the NC samples were not used for further deployments.

The biofouling processing involved sieving the samples gently through a 0.5 mm mesh sieve. The organisms retained were then identified, counted, and weighed (fresh weight). Taxonomy for macroinvertebrates and macroalgae was done to the lowest taxonomic level possible and was standardized in accordance to the World Register of Marine Species ( WoRMS) and the AlgaeBase, respectively.

In parallel to biofouling sampling, seawater temperature (°C), salinity, dissolved oxygen (DO; mg L ^-1^) and total chlorophyll (Chl.; µg L ^-1^) were measured at 3 m depth using a YSI ProDSS handheld multiparameter probe. With no particular reason, a greater number of measurements coincided with low tides (spring: two out of two sampling events; summer: three out of six sampling events; autumn: three out of five sampling events).

### Data analysis

All statistical analyses were performed with PRIMER 6 + PERMANOVA software ( Anderson *et al.,* 2008; Clarke & Gorley, 2006). The PERMANOVA, SIMPER, and PCO analyses can be performed using open-source software such as R (using the Vegan package in R). A PRIMER trial version can be downloaded from the PRIMER website. PERMANOVA is a non-parametric method based on permutation tests that can be used both for univariate and multivariate data, being especially useful when sample sizes are small or there are unequal groups sizes, which is the case of this research.

***Seawater parameters.*** For each seawater parameter (temperature, salinity, DO, and Chl.), statistically significant differences among seasons were tested using permutational multivariate analysis of variance (PERMANOVA). The design included one fixed factor, ‘Season’ (three levels: spring, summer, and autumn). The Euclidean distance was used in the calculation of the resemblance matrix. The statistical significance of variance components was tested using 999 permutations and unrestricted permutation of raw data, with a significance level of α = 0.05.

***Biofouling parameters.*** Prior to data analysis, macroinvertebrate density was standardized to number of individuals per square metre (ind m ^-2^), and invertebrate and algae biomass were standardized to grams of fresh weight per square metre (g FW m ^-2^).

Six biofouling parameters were used to describe the biofouling communities. Four were univariate parameters: number of taxa ( *Richness*), total biofouling biomass ( *TBiom*), total biofouling density ( *TDens*) and *Thickness*, and two were multivariate parameters: individual organisms’ biomass ( *BIOM*) and density ( *DENS*).

For statistical analysis of biofouling data, it was first assessed the feasibility of using the data of both cylinder treatments – LC and NC – together in subsequent analyses. Statistically significant differences between the two treatments were tested using PERMANOVA applied individually to *Richness*, *TBiom*, *BIOM, TDens, DENS*, and *Thickness*. The statistical design included the fixed factors ‘Treatment’ (two levels: LC and NC), ‘Season’ (three levels: spring, summer, and autumn) and ‘Submersion’ (seven levels: 1, 2, 3, 4, 5, 6 and 8W) nested in ‘Season’. The Euclidean distance (univariate data) or Bray Curtis similarity (multivariate data) were used in the calculation of resemblance matrices, with addition of a dummy variable of the lowest value in the source data matrix. Before calculating the resemblance matrices, *TBiom, TDens, BIOM* and *DENS* data were square root-transformed to reduce the influence of naturally abundant organisms ( *e.g.* barnacles) in the analyses. The statistical significance of variance components was tested using 999 permutations, with unrestricted permutation of raw data (univariate data) or permutation of residuals under a reduced model (multivariate data), with a significance level of α = 0.05. When the possible permutations were <100 the Monte Carlo *p* value was selected.

Afterwards, using the LC and NC data combined (because no statistical differences were previously found; see *Extended data*), statistical differences among seasons and among submersion periods within each season were assessed individually for *Richness*, *TBiom*, *BIOM, TDens, DENS,* and *Thickness*. The statistical design included the factors ‘Season’ and ‘Submersion’ nested in ‘Season’, and the same options were used as for the previous PERMANOVA.

Following this, analysis of similarity percentages (SIMPER) was applied individually to *BIOM* and *DENS* to identify the taxa which contributed mostly to the statistical differences. First, dissimilarities among seasons were assessed using two-way crossed designs with factors ‘Season’ and ‘Submersion’. Then, dissimilarities among submersion periods within each season were assessed selecting each season data and using a one-way design with the factor ‘Submersion’. For all the SIMPER analyses a 90% cut-off was used, with square-root transformation of data.

***Relationship between the biofouling and seawater parameters.*** To visualize the seasonal relation between biofouling parameters ( *Richness, TBiom, TDens,* and *Thickness*) and seawater parameters (temperature, salinity, DO, and Chl.) a principal coordinates analysis (PCO) was conducted. To do this, the seawater parameters data were averaged per season and that value was used for each biofouling sample in that season ( *e.g.* spring water temperature was the same for the spring biofouling 1W, 2W, and 4W samples).

***Relationship between frictional resistance forces and biofouling data.*** The number of frictional resistance measurements along a cylinder depended on the velocity of scraping (faster scraping resulting in fewer measurements). As this was a first version of the scraping system, the measurement of scrapings velocity was not possible.

Before assessing the relationship between frictional resistance and biofouling data, the prior was pre-processed. Firstly, outliers were removed, for example, associated with the acceleration at the start or deceleration at the stop of the scraping event owed to the tightness of the plastic scraper to the samples. Then, mean values and standard deviation were calculated using all the samples within a submersion period within a season. For example, the mean ± standard deviation of the sample “summer 1W” was calculated using the measurements of the three “1W” samples of “summer”. Using this frictional resistance data **, s**tatistical differences among seasons and among submersion periods within season were assessed with PERMANOVA. The same options as for the previous PERMANOVA of univariate data were used.

The relationships between the frictional resistance forces data and biofouling parameters ( *Richness, TBiom, BIOM, TDens, DENS,* and *Thickness*), as well as among biofouling parameters, were calculated with RELATE (comparative Mantel-type tests on similarity matrices). The resemblance matrices for the friction forces and the biofouling parameters (all of which are univariate data) were calculated as previously for PERMANOVA. To match all resemblances matrices, samples without frictional resistance data were removed from the biofouling data matrices prior to calculating the resemblance matrices. After, Pearson correlations between the frictional resistance forces data and biofouling parameters ( *Richness*, *TBiom*, *TDens*, and *Thickness*) were calculated.

## Results

**Figure 4.**
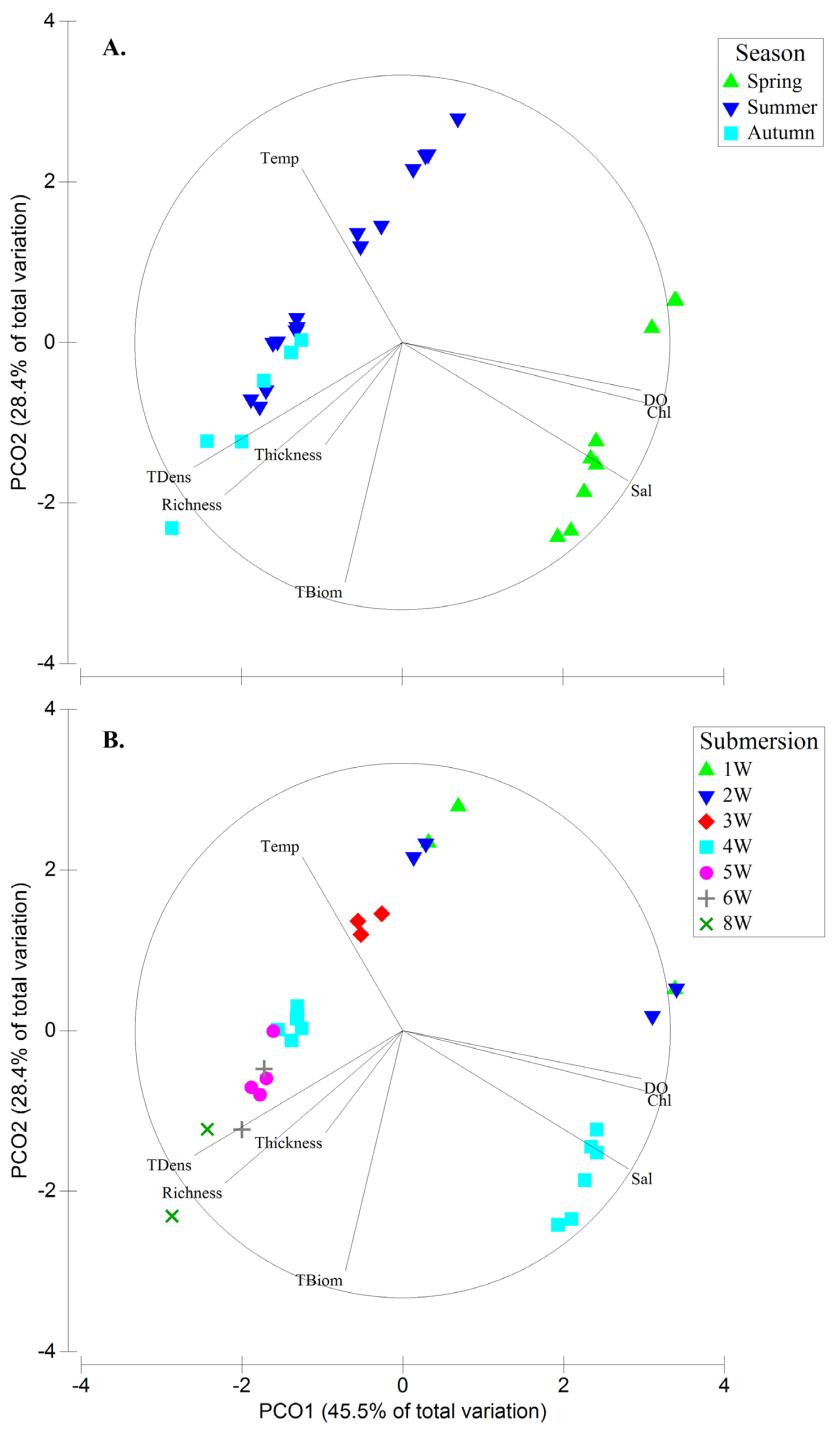
Principal Coordinates analysis (PCO) plot showing trends of seawater parameters (temperature, salinity, dissolved oxygen [DO] and total chlorophyll [Chl.]) and biofouling parameters ( *Richness, TBiom, TDens,* and *Thickness*) among seasons (A.) and submersion periods (B.).

Mean water temperature was higher in summer (18.5 ± 1.2 ºC), whereas mean salinity, DO, and Chl. were higher in spring (40.6 ± 0.1, 7.23 ± 0.29 mg L ^-1^, and 2.63 ± 0.73 µg L ^-1^ respectively ( Table 2A., Figure 4A.). Statistically significant differences were found among seasons for all parameters except DO ( *Extended data*).

**Table 2.** Seasonal values for the seawater parameters (A.) and the biofouling parameters (B.). Mean ± standard deviation are presented, except for Richness. Greater mean values among seasons are highlighted in black (white font). Greater mean values among submersion periods within season are presented in bold.

A.	Temperature (°C)	Salinity	Dissolved Oxygen (mg L ^-1^)	Chlorophyll (µg L ^-1^)
**Spring**	16.8 ± 0.2	40.6 ± 0.1	7.23 ± 0.29	2.63 ± 0.73
**Summer**	18.5 ± 1.2	38.6 ± 1.1	6.83 ± 0.68	1.50 ± 1.13
**Autumn**	16.7 ± 1.6	38.9 ± 1.2	6.84 ± 0.21	0.76 ± 0.28
B.	*Richness* (total no. taxa)	*TBiom* (g FW m ^-2^)	*TDens* (ind m ^-2^)	*Thickness* (mm)
**Spring**	16	23.9 ± 19.5	352.0 ± 280.9	0.78 ± 0.45
**1W**	3	0.03 ± 0.00	15.3 ± 0.0	0.20 ± 0.00
**2W**	7	0.71 ± 0.40	38.3 ± 54.1	0.25 ± 0.21
**4W**	**16**	**35.6 ± 10.6**	**512.6 ± 180.7**	**1.05 ± 0.22**
**Summer**	21	10.4 ± 10.3	1330.4 ± 1225.0	1.06 ± 0.87
**1W**	5	0.09 ± 0.16	12.1 ± 20.9	0.11 ± 0.18
**2W**	9	0.58 ± 0.14	63.3 ± 12.8	0.10 ± 0.00
**3W**	17	5.7 ± 3.3	482.3 ± 297.4	0.76 ± 0.55
**4W**	**20**	13.1± 2.5	2224.6 ± 478.0	1.65 ± 0.66
**5W**	**20**	**23.8 ± 8.8**	**2694.8 ± 373.6**	**1.90 ± 0.26**
**Autumn**	20	33.9 ± 13.5	2966.1 ± 1759.6	0.43 ± 0.28
**4W**	16	21.6 ± 0.31	1039.9 ± 217.4	**0.49 ± 0.01**
**6W**	15	35.6 ± 15.1	2929.9 ± 511.5	0.44 ± 0.47
**8W**	**20**	**44.4 ± 12.6**	**4928.4 ± 217.4**	0.37 ± 0.37

### Biofouling parameters

**Figure 5.**
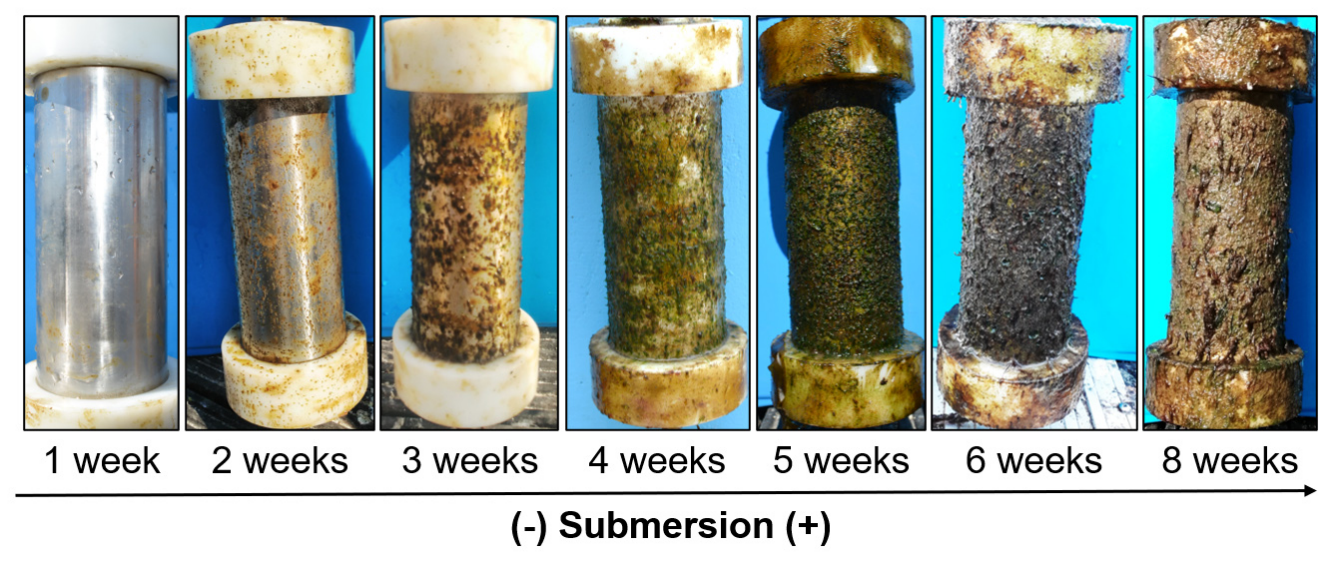
Biofouling growth after one, two, three, four, five, six, and eight weeks of samples submersion.

The biofouling growth was noticeable between seasons and in each season with increasing submersion time of samples ( Figure 4, Figure 5, Table 2B., Table 3). Strong statistically significant relationships (RELATE Rho≥0.70) ( Table 4A.) were found among all the biofouling parameters, as well as high positive correlations among *Richness, TBiom* (total biomass), and *TDens* (total density) (≥0.70) ( Table 4B.).

*Richness, TBiom* and *TDens* registered higher mean values in each season at the longest submersion period, with higher values calculated for autumn at 8W. Mean *Thickness* increased with increasing submersion in spring and summer ( Table 2B.). Statistically significant differences were found among seasons for *Richness* (all seasons) *, TBiom* (except between spring-summer) *, BIOM* (all seasons) *, TDens* (all seasons) *, DENS* (all seasons), and *Thickness* (except between spring-autumn). Simultaneously, for all the parameters above, statistical differences were found among submersion periods in spring and summer; in autumn, statistical differences between submersion periods were found for *TDens* and *DENS* ( *Extended data*).

**Table 3.** List of taxa found in this study, showing their presence (in grey) across submersion periods (1–8W) within each season. Total macroalgal and macroinvertebrate taxa are presented per submersion period and per season at the bottom. The number of occurrences (occ.) of each taxon in the study is shown on the right side. Greater numbers are presented in bold. N.I.: Not identified.

Group			Taxa	Spring			Summer					Autum			Occ.
				1W	2W	4W	1W	2W	3W	4W	5W	4W	6W	8W	
**Macroalgae**	Ph. Chlorophyta	Or. Ulvales	*Ulva* sp. (tubular-like form)												**10**
			*Ulva* sp. (leaf-like form)												**10**
	Ph. Rhodophyta	Or. Ceramiales	cf. *Tiffaniella capitata*												7
			cf. *Pterothamnion crispum*												5
			*Polysiphonia* sp.												**8**
			cf. *Halurus flosculosus* / *Bornetia secundiflora*												7
		Ph. Rhodophya	Rhodophyta N.I.												**8**
	Cl. Phaeophyceae	Or. Ectocarpales / Or. Sphacelariales	*Hincksia* sp. / *Sphacelaria* sp.												**9**
**Macroalgal taxa per submersion period**				1	4	**9**	1	7	8	**9**	**9**	**9**	7	**9**	
**Macroalgal taxa per season**				**9**			**9**					**9**			
**Macroinvertebrates**	Ph. Bryozoa	Ph. Bryozoa	Bryozoa N.I.												**8**
	S.Ph. Crustacea	Or. Amphipoda	Amphipoda N.I.												**9**
			*Caprella equilibra*												7
		Or. Decapoda	Anomura / Brachyura N.I.												3
			cf. *Pasiphaea sivado*												5
		Or. Isopoda	Gnathiidae N.I.												2
			*Tanais dulongii*												7
		Or. Sessilia	Barnacles ( *Perforatus perforatus,* *Amphibalanus amphitrite,* *Austrominius modestus*)												**10**
	Cl. Pycnogonida	Or. Pantopoda	*Ammothella longipes*												1
	Cl. Polychaeta	F. Serpulidae	*Spirobranchus* sp.												7
		F. Syllidae	Syllidae N.I.												2
	Ph. Mollusca	F. Nereididae	Nereididae N.I.												1
		Cl. Bivalvia	*Mytillus galloprovincialis*												2
		Cl. Gastropoda	cf. *Crisilla semistriata*												1
**Macroinvertebrate taxa per submersion period**				2	3	**7**	4	2	9	**11**	**11**	7	8	**11**	
**Macroinvertebrate taxa per season**				7			**12**					11			
**Total taxa per submersion period**				3	7	**16**	5	9	17	**20**	**20**	16	15	**20**	
**Total taxa per season**				16			**21**					20			

**Table 4.** Relationships between the frictional resistance forces and biofouling parameters. A. RELATE analysis; stronger relations (Rho≥0.70) are presented in bold. B. Pearson correlations; higher correlations (≥0.50) are presented in bold.

A.	*Richness*	*TBiom*	*BIOM*	*TDens*	*DENS*	*Thickness*
**Friction forces**	0.396	0.237	0.382	0.248	0.380	0.094 *
***Richness***		**0.696**	**0.824**	0.674	**0.845**	0.179
***TBiom***			**0.742**	**0.790**	**0.784**	0.118 *
***BIOM***				0.668	**0.898**	0.104 *
***TDens***					**0.803**	0.201
***DENS***						0.255
B.	*Richness*	*TBiom*	*TDens*	*Thickness*		
**Friction forces**	**-0.722**	-0.401	**-0.525**	**-0.532**		
***Richness***		**0.698**	**0.715**	**0.640**		
***TBiom***			**0.857**	0.192		
***TDens***				0.368		

* Not statistically significant (signiﬁcance level of α = 0.05)

In total 24 taxa were found, 9 macroalgal taxa and 15 macroinvertebrate taxa ( Table 3). Summer and autumn registered more taxa (21 and 20 taxa, respectively), which in each season generally increased with the submersion period (maximum of 20 taxa in summer 4W, summer 5W, and autumn 8W) ( Table 2B., Table 3).

All macroalgal taxa in this study were recorded in every season, whereas more macroinvertebrate taxa (12 taxa) were found during summer. The most frequent taxa were the green algae *Ulva* sp. (both tubular-like and leaf-like forms) and barnacles (including *Perforatus perforatus, Amphibalanus amphitrite,* and the NIS *Austrominius modestus*) (10 occurrences each), followed by the brown algae *Hincksia* sp./ *Sphacelaria* sp. and Amphipoda N.I. (9 occurrences each), the red algae *Polysiphonia* sp. and Rhodophyta N.I., and Bryozoa N.I. (8 occurrences each). Some taxa were found only in summer (Anomura/Brachyura N.I., Gnathiidae N.I., Syllidae N.I., and *Mytillus galloprovincialis*) or in autumn ( *Ammothella longipes*, cf. *Crisilla semistriata*, and Nereididae N.I.) ( Table 3). Overall, some species succession in the colonization process was observed. For example, after one week of submersion, only the opportunistic green algae ( *Ulva* spp.), barnacles (cf. *P. perforatus/A. amphitrite* and *A. modestus*), bryozoans (Bryozoa N.I.; in summer) and other crustaceans (Anomura/Brachyura N.I.; in summer) were recorded; after two weeks, filamentous brown algae ( *Hincksia* sp./ *Sphacelaria* sp.), red algae ( *e.g.* from the order Ceramiales; in summer) and many amphipod individuals were observed; after three or more weeks, several other macroalgal and macroinvertebrate taxa joined the biofouling communities ( Table 3).

**Figure 6.**
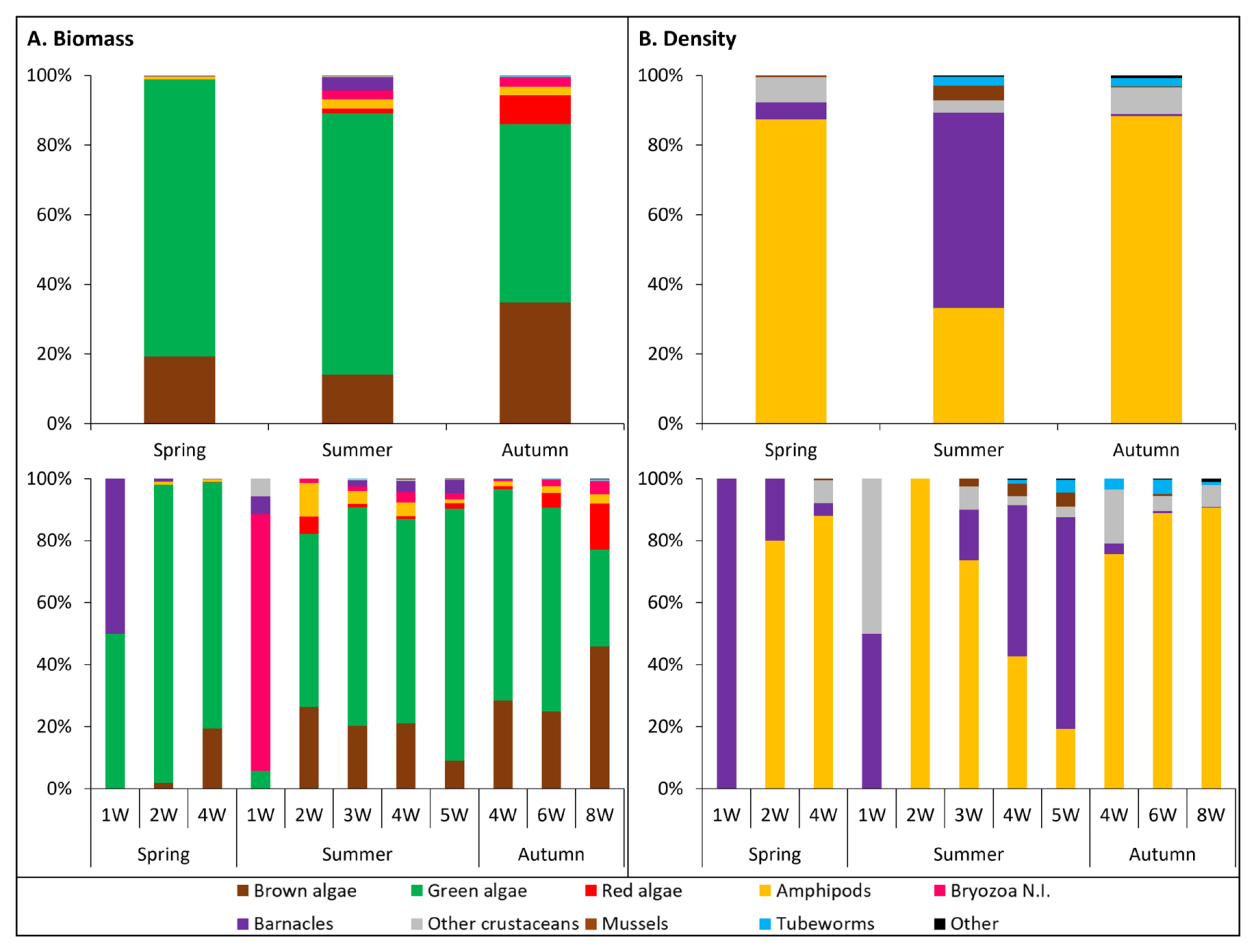
Contribution of major biofouling groups to the communities’ biomass (A.) and density (B.) among seasons (top) and submersion periods (bottom).

Not only the structure of the biofouling communities changed between seasons and between submersion periods in each season, so did the proportion of biofoulers biomass and density to the total ( Figure 6):

Regarding the biofoulers biomass ( Figure 6A.), in all seasons the green and brown algae accounted for the greatest share of the total (51–79% and 14–35%, respectively), followed by red algae (1% in summer, 8% in autumn), amphipods (3% in summer, 2% in autumn), bryozoans (3% in summer, 6% in autumn), and barnacles (4% in summer). Changes in the taxa and their proportions between submersion periods in each season were evident, and also between seasons at the same submersion period. Below are provided details for the submersion periods assessed in more than one season:

**• 1W (spring vs. summer):** In spring, only green algae and barnacles were found, each accounting for 50% of the total biomass. In summer, more taxa (bryozoans, Anomura/Brachyura N.I.) were part of the assemblages compared to spring; green algae and barnacles presented much lower proportions (6% each) and bryozoans accounted for 83%.

**• 2W (spring vs. summer):** In spring, brown algae and amphipods joined the assemblages; the green algae accounted for 96% of the total biomass. In summer, brown algae, red algae, and amphipods were also part of the assemblages; bryozoans had a very much reduced proportion (1%), whereas the proportion of green algae, brown algae, and amphipods increased (to 56%, 26%, and 11%, respectively).

**• 4W (spring vs. summer vs. autumn:** The assemblages were composed of most of the taxa found in this study, especially in summer and autumn ( *e.g.* bryozoans were not registered in spring). In all seasons the green algae accounted for greater proportion (66–68% in summer and autumn to 79% in spring), followed by brown algae (19% in spring to 28% in autumn). Other taxa accounted for very low proportions, with the higher values being observed in summer (amphipods and barnacles: 4% each, bryozoans: 3%, red algae: 1%).

Concerning to other submersion periods ( *i.e.* summer 3W and 5W, and autumn 6W and 8W), the distribution of taxa proportion generally followed a similar order compared to the 4W, with green algae accounting for the greatest proportion, followed by brown algae, amphipods, barnacles in summer, bryozoans, and red algae.

Regarding the biofoulers density ( Figure 6B.), in all seasons amphipods (33% in summer to 87–88% in spring-autumn), barnacles (1% in autumn to 56% in summer), and other crustaceans (3% in spring to 7–8% in spring-autumn) contributed the most to the total. Like for the biomass, the taxa and their proportions to the total density changed between submersion periods in each season and between seasons at the same submersion period. Below are provided details for the submersion periods assessed in more than one season:

**• 1W (spring vs. summer):** As mentioned above for the biomass, in spring only green algae and barnacles were found. Thus, barnacles accounted for 100% of the density. In summer, barnacles and Anomura/Brachyura N.I. each accounted for 50%.

**• 2W (spring vs. summer):** At 2W the amphipods achieved much greater expression. In spring, they accounted for 80% of the total density and barnacles accounted for the remaining 20%. In summer, amphipods accounted for 100% of the total density.

**• 4W (spring vs. summer vs. autumn:** The amphipods generally accounted for the greatest proportions in all seasons (43% in summer to 88% in spring), followed by barnacles (3–4% in spring-autumn to 49% in summer), motile crustaceans (3% in summer to 17% in autumn), mussels (4% in summer), and tubeworms (1% in summer to 3% in autumn).

Like for the biomass, at other submersion periods ( *i.e.* summer 3W and 5W, and autumn 6W and 8W) the distribution of taxa proportion generally follows a similar order compared to the 4W, with barnacles and amphipods accounting for the greatest proportions, followed by mussels, tubeworms, and motile crustaceans, in summer, and amphipods accounting for the greatest proportions, followed by motile crustaceans and tubeworms, in autumn.

The SIMPER analyses (cut-off 90%) provided further insights about the communities’ structure, with similar patterns being found using *BIOM* or *DENS* ( *Extended data*). The similarities within the seasons were high, ranging between 68.59% ( *BIOM*) / 67.53% ( *DENS)* in summer and 85.66% ( *BIOM*) / 80.44% (DENS) in autumn. The greatest dissimilarities between seasons were found between spring-summer, 44.47% using *BIOM* and 55.1% using *DENS*.

In each season, similarities within the submersion periods were also high. Using *BIOM*, similarities ranged between 60.20% for spring 2W and 94.41% for autumn 4W. Using *DENS*, similarities ranged between 54.6% for summer 3W and 92.92% for summer 2W. Dissimilarities between submersion periods were also high, generally greater between the submersion periods farther from each other, *i.e.* in spring between 1W-4W (95.47% using *BIOM* and 79.85% using *DENS*), in summer between 1W-5W (95.61% using *BIOM* and 97.70% using *DENS*; second to 1W-2W: 96.82% and 100%, respectively) and in autumn between 4–8W (34.58% using *BIOM* and 43.38% using *DENS*). The lowest dissimilarities were observed between adjacent submersion periods, *i.e.* in spring between 1–2W (74.50%) using *BIOM* and between 2W–4W (74.01%) using *DENS*, in summer between 4–5W (25.93% using *BIOM* and 23.15% using *DENS*) and in autumn between 6–8W (21.69% using *BIOM* and 24.72% using *DENS*) ( *Extended data*).

**Figure 7.**
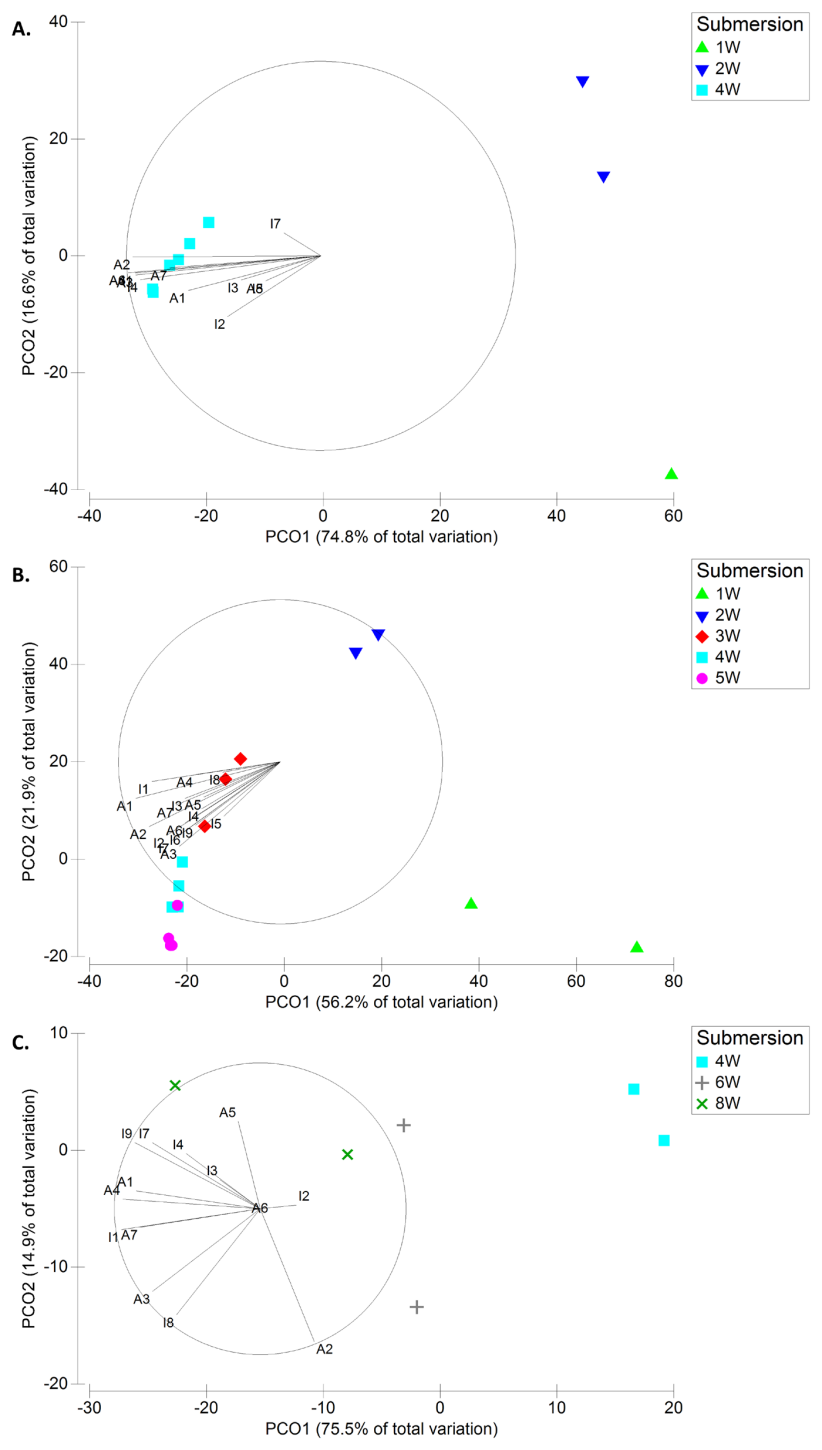
Principal Coordinates Ordination (PCO) plots of the taxa contributing the most to dissimilarities (from SIMPER) using biomass data (BIOM) in Spring (A.), Summer (B.), and Autumn (C.). Taxa key – Algae: A1- *Hincksia* sp./ *Sphacelaria* sp.; A2- *Ulva* sp. (tubular-like form); A3- *Ulva* sp. (leaf-like form); A4- *Polysiphonia* sp.; A5-cf. *T. capitata*; A6-cf. *H. flosculosus*/ *B. secundiflora*; A7-Rhodophyta N.I.. Invertebrates: I1-Amphipoda N.I.; I2-Barnacles; I3- *C. equilibra*; I4- *T. dulongii*; I5-Anomura/Brachyura N.I.; I6- *M. galloprovincialis*; I7- *Spirobranchus* sp.; I8-cf. *P. sivado*; I9-Bryozoa N.I.

**Figure 8.**
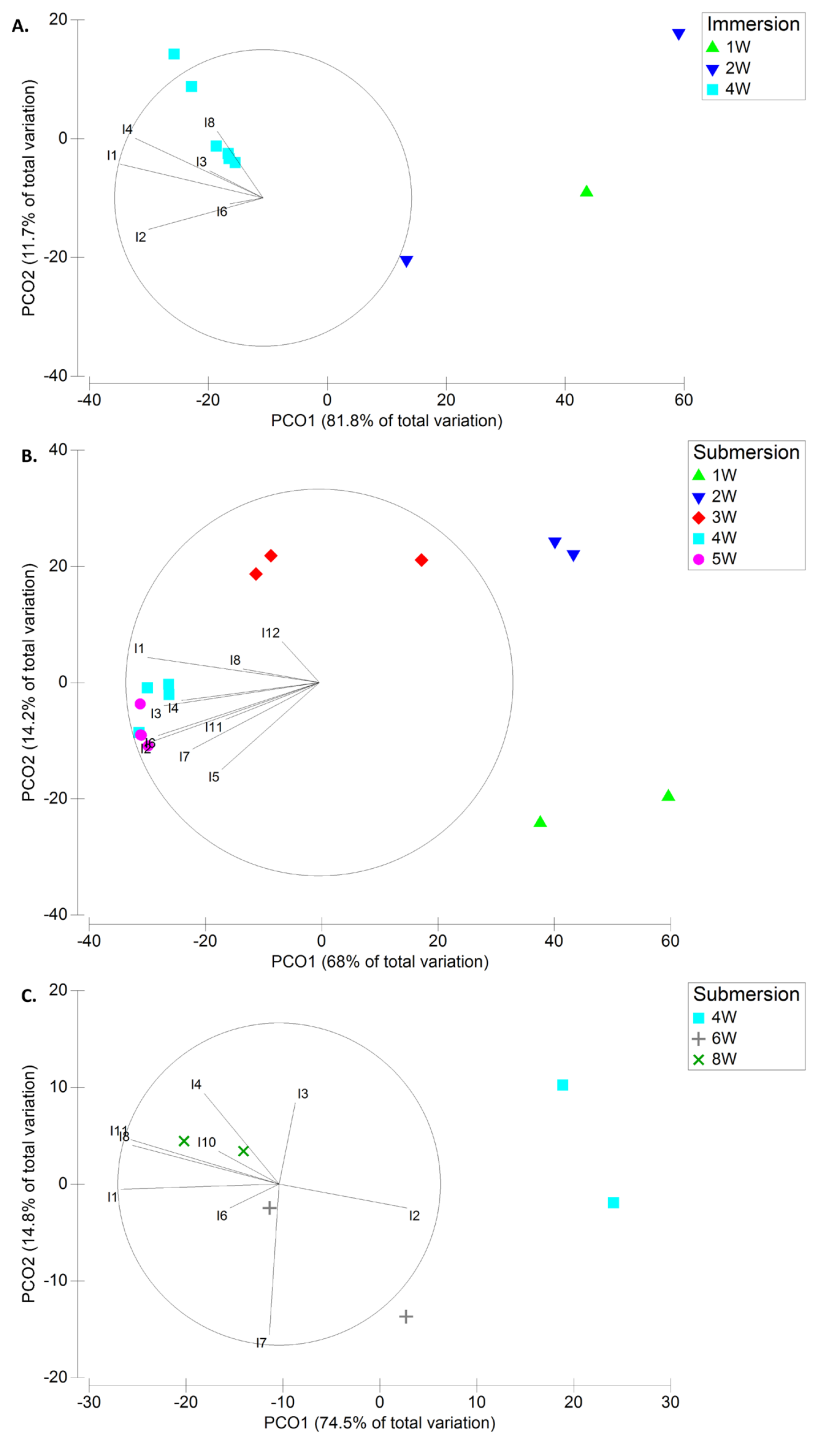
Principal Coordinates Ordination (PCO) plots of the taxa contributing the most to dissimilarities (from SIMPER) using density data (DENS) in Spring (A.), Summer (B.), and Autumn (C.). Taxa key – Invertebrates: I1-Amphipoda N.I.; I2-Barnacles; I3- *C. equilibra*; I4- *T. dulongii*; I5-Anomura/Brachyura N.I.; I6- *M. galloprovincialis*; I7- *Spirobranchus* sp.; I8-cf. *P. sivado*; I10- *A. semistriata*; I11-Syllidae N.I.; I12-Gnathiidae N.I..

Sixteen taxa (7 macroalgal + 9 macroinvertebrate taxa) were responsible for the dissimilarities regarding *BIOM*, while 11 macroinvertebrate taxa were main contributors to the dissimilarities regarding *DENS*. Most of the taxa, and especially the Amphipoda N.I., registered biomass or density increase with increasing submersion period in each season and registered higher mean values in autumn ( Figure 7, Figure 8; Table 5). Some exceptions were the barnacles and mussels ( *M. galloprovincialis*) which registered much greater biomass and density in summer, and the opportunistic green algae *Ulva* spp. which were the only taxa with greater biomass in spring.

**Table 5.** Biomass (g FW m ^-2^) (A.) and density (ind m ^-2^) (B.) of the main taxa contributing to similarities within and dissimilarities among seasons and among submersion periods within the seasons (from SIMPER analysis). Mean ± standard deviation values are presented. Grey colour identifies increasing values with increasing submersion period in each season.

A1. Macroalgae biomass		*Hincksia* sp. / *Sphacelaria* sp.	*Ulva* sp. (tubular-like form)	*Ulva* sp. (leaf-like form)	cf. *Polysiphonia* sp.	cf. *T.* *capitata*	cf. *H.* *flosculosus* / B. secundiflora	Rhodophyta N.I.				
**Spring**		4.62 ± 5.43	**15.55 ± 11.86**	**3.45 ± 2.91**	0.009 ± 0.006	0.002 ± 0.004	0.009±0.007	0.008 ± 0.007				
**Summer**		1.46 ± 1.19	6.58 ± 6.97	1.20 ± 1.79	0.019 ± 0.024	**0.069 ± 0.138**	0.012±0.018	0.044 ± 0.067				
**Autumn**		**11.79 ± 8.28**	14.61 ± 5.64	2.71 ± 2.10	**2.63 ± 2.74**	0.005 ± 0.008	**0.016±0**	**0.162 ± 0.126**				
**Spring**	**1W**	0 ± 0	0.014 ± 0	0±0	0 ± 0	0 ± 0	0±0	0 ± 0				
	**2W**	0.014 ± 0	0.65 ± 0.33	0.031 ± 0.031	0 ± 0	0 ± 0	0±0	0 ± 0				
	**4W**	6.92 ± 5.32	23.11 ± 6.30	5.17 ± 1.96	0.014 ± 0	0.002 ± 0.005	0.014±0	0.011 ± 0.005				
**Summer**	**1W**	0 ± 0	0 ± 0	0.005 ± 0.008	0 ± 0	0 ± 0	0±0	0 ± 0				
	**2W**	0.15 ± 0.03	0.32 ± 0.05	0.008 ± 0.008	0.008 ± 0.008	0.016 ± 0	0±0	0.008 ± 0.008				
	**3W**	1.16 ± 0.67	3.95 ± 2.93	0.066 ± 0.035	0.030 ± 0.031	0.016 ± 0	0.005±0.009	0.016 ± 0				
	**4W**	2.75 ± 0.67	8.58 ± 2.69	0.500 ± 0.579	0.005 ± 0.030	0.035 ± 0.027	0.026±0.032	0.035 ± 0.024				
	**5W**	2.15 ± 0.40	15.37 ± 6.65	3.92 ± 1.46	0.026 ± 0.016	0.23 ± 0.20	0.016±0	0.13 ± 0.08				
**Autumn**	**4W**	6.14 ± 0.53	14.59 ± 0.79	0.154 ± 0.009	0.14 ± 0.03	0.008 ±0.008	0.016±0	0.016 ± 0				
	**6W**	8.87 ± 2.43	18.29 ± 7.75	5.09 ± 0.93	1.38 ± 0.10	0 ± 0	0.016±0	0.27 ± 0.09				
	**8W**	20.37 ± 9.25	10.94 ± 2.80	2.89 ± 0.18	6.37 ± 0.87	0.008 ± 0.008	0.016±0	0.20 ± 0.07				
A2. Macroinvertebrates biomass		Amphipoda N.I.	Barnacles	*C. equilibra*	*T. dulongii*	Anomura / Brachyura N.I.	*M. * *galloprovincialis*	*Spirobranchus* sp.	cf. *P. sivado*	Bryozoa N.I.		
**Spring**		0.20 ± 0.16	0.033 ± 0.045	0.003±0.006	0.013±0.014	0±0	0±0	0.002±0.005	0.007±0.020	0 ± 0		
**Summer**		0.26 ± 0.25	**0.401 ± 0.469**	0.012±0.014	0.014±0.027	**0.013±0.027**	**0.007±0.008**	0.008±0.008	0.004±0.007	0.26 ± 0.34		
**Autumn**		**0.80 ± 0.42**	0.014 ± 0.006	**0.024±0.034**	**0.042±0.061**	0±0	0±0	**0.092±0.139**	**0.014±0.021**	**0.93 ± 1.02**		
**Spring**	**1W**	0 ± 0	0.014 ± 0	0±0	0±0	0±0	0±0	0±0	0±0	0 ± 0		
	**2W**	0.007 ± 0.007	0.007 ± 0.007	0±0	0±0	0±0	0±0	0±0	0±0	0 ± 0		
	**4W**	0.30 ± 0.09	0.045 ± 0.051	0.005±0.008	0.020±0.013	0±0	0±0	0.002±0.006	0.010±0.025	0 ± 0		
**Summer**	**1W**	0 ± 0	0.005 ± 0.008	0±0	0±0	0.005±0.009	0±0	0±0	0±0	0.08 ± 0.11		
	**2W**	0.063 ± 0.009	0 ± 0	0±0	0±0	0±0	0±0	0±0	0±0	0.008 ± 0.008		
	**3W**	0.22 ± 0.17	0.11 ± 0.12	0.011±0.009	0.011±0.009	0±0	0±0	0.005±0.009	0.005±0.009	0.09 ± 0.07		
	**4W**	0.50 ± 0.25	0.61 ± 0.36	0.017±0.015	0.008±0.009	0.035±0.049	0.016±0	0.012±0.008	0.012±0.008	0.33 ± 0.38		
	**5W**	0.27 ± 0.07	1.04 ± 0.26	0.030±0.010	0.054±0.049	0.011±0.009	0.011±0.009	0.016±0	0±0	0.46 ± 0.40		
**Autumn**	**4W**	0.32 ± 0.05	0.016 ± 0	0.018±0.001	0.008±0.012	0±0	0±0	0.016±0	0±0	0.16 ± 0		
	**6W**	0.78 ± 0.13	0.016 ± 0	0.008±0.012	0.018±0.026	0±0	0±0	0.071±0.078	0.027±0.038	0.73 ± 0.55		
	**8W**	1.31 ± 0.06	0.008 ± 0.008	0.045±0.064	0.099±0.090	0±0	0±0	0.189±0.244	0.016±0	1.88 ± 1.12		
B. Macroinvertebrates density		Amphipoda N.I	Barnacles	*C. equilibra*	*T. dulongii*	Anomura / Brachyura N.I.	*M.* *galloprovincialis*	*Spirobranchus* sp.	cf. *P. sivado*	*A.* *semistriata*	Syllidae N.I.	Gnathiidae N.I.
**Spring**		297.6 ± 228.6	17.0 ± 8.7	10.2 ± 20.4	23.8 ± 24.0	0 ± 0	1.7 ± 4.8	0 ± 0	1.7±5.1	0±0	0±0	0±0
**Summer**		410.3 ± 387.3	**747.2 ± 833.8**	**31.7 ± 39.2**	26.0 ± 43.4	**12.4 ± 18.9**	**55.4 ± 71.9**	35.0 ± 63.8	4.5±8.1	0±0	4.5±8.1	**3.4±9.8**
**Autumn**		**2592.3 ± 1512.0**	21.1 ± 16.2	27.1 ± 30.9	**217.0 ± 170.0**	0 ± 0	6.0 ± 13.5	**72.3 ± 66.0**	**9.0±9.9**	**3.0±7.4**	**12.1±14.8**	0±0
**Spring**	**1W**	0 ± 0	15.3 ± 0	0 ± 0	0±0	0 ± 0	0 ± 0	0 ± 0	0±0	0±0	0±0	0±0
	**2W**	30.6 ± 30.6	7.7 ± 7.7	0 ± 0	0±0	0 ± 0	0 ± 0	0 ± 0	0±0	0±0	0±0	0±0
	**4W**	436.1 ± 142.7	20.4 ± 7.2	15.3 ± 23.4	35.7 ± 21.0	0 ± 0	2.6 ± 5.7	0 ± 0	2.6±6.2	0±0	0±0	0±0
**Summer**	**1W**	0 ± 0	6.0 ± 8.5	0 ± 0	0±0	6.0 ± 8.5	0 ± 0	0 ± 0	0±0	0±0	0±0	0±0
	**2W**	63.3 ± 9.0	0 ± 0	0 ± 0	0 ± 0	0 ± 0	0 ± 0	0 ± 0	0±0	0±0	0±0	0±0
	**3W**	343.6 ± 227.3	78.4 ± 30.7	12.1 ± 8.5	18.1 ± 14.8	0 ± 0	12.1 ± 17.1	0 ± 0	6.0±10.4	0±0	0±0	12.1±20.9
	**4W**	729.5 ± 343.4	1386.6 ± 559.3	30.1 ± 27.1	12.1 ± 12.8	36.2 ± 26.7	102.5 ± 44.3	18.1 ± 20.2	13.6±9.0	0±0	9.0±10.4	0±0
	**5W**	447.6 ± 89.9	1840.2 ± 362.7	72.3 ± 44.3	72.3 ± 63.9	13.6 ± 7.8	122.1 ± 87.1	113.0 ± 85.2	0±0	0±0	9.0±10.4	4.5±9.0
**Autumn**	**4W**	759.6 ± 18.1	36.2 ± 0	27.1 ± 9.0	180.9 ± 144.7	0 ± 0	0 ± 0	36.2 ± 18.1	0±0	0±0	0±0	0±0
	**6W**	2595.3 ± 388.8	18.1 ± 18.1	9.0 ± 9.0	135.6 ± 27.1	0 ± 0	18.1 ± 18.1	135.6 ± 81.4	9.0±12.8	0±0	9.0±12.8	0±0
	**8W**	4422.0 ± 27.1	9.0 ± 9.0	45.2 ± 45.2	334.6 ± 208.0	0 ± 0	0 ± 0	45.2 ± 9.0	18.1±0	9.0±12.8	27.1±12.8	0±0

### Frictional resistance forces and its relationship with biofouling data

**Figure 9.**
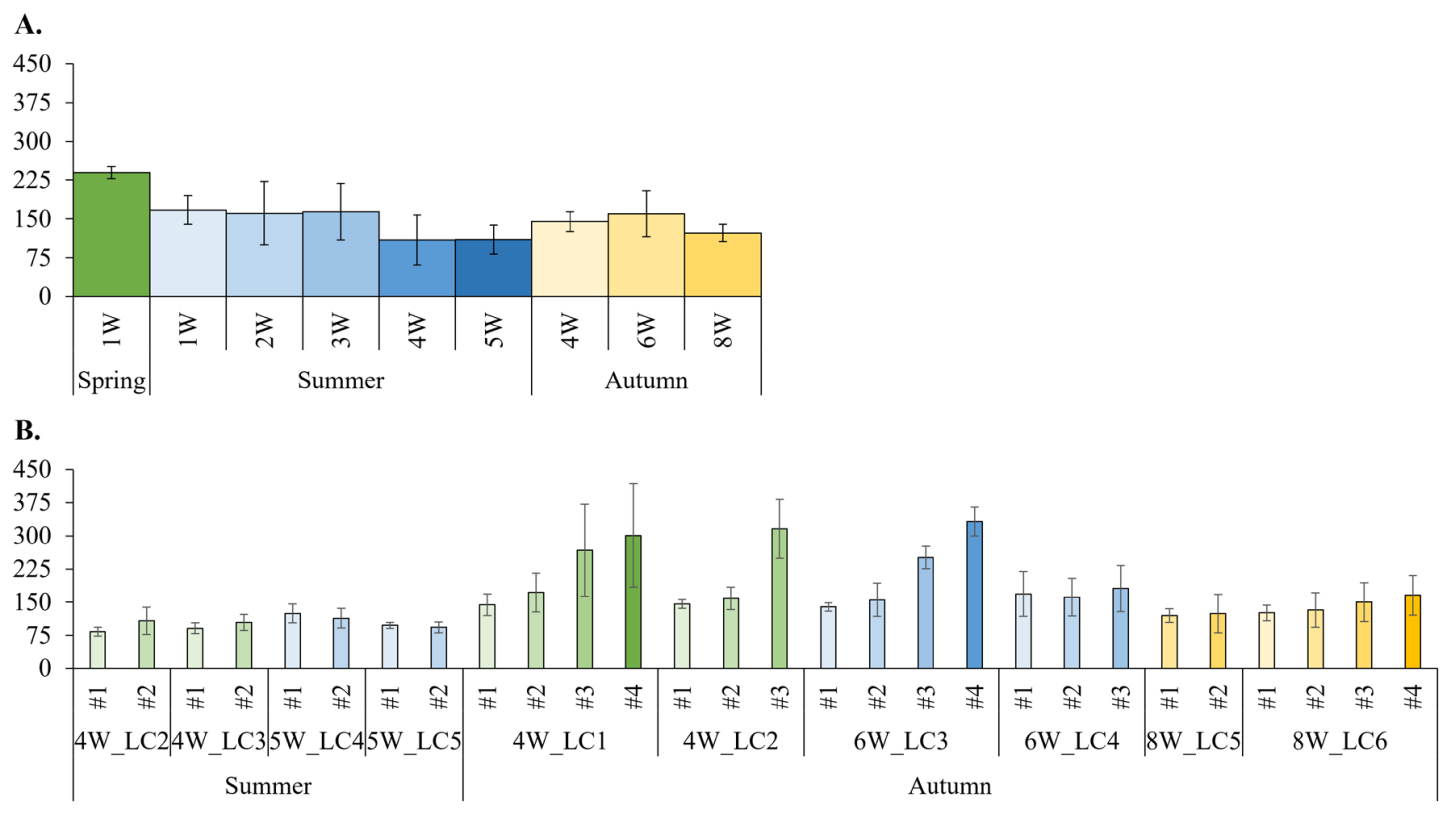
A. Frictional resistance forces in different submersion periods (1–8W) within each season; increasing colours darkness reflect increasing submersion periods. **B**. Frictional resistance forces from subsequent scrapings (#1 to #4) of some summer and autumn samples; increasing colours darkness reflect increasing scraping #. Mean ± standard deviation values are presented.

Mean frictional resistance forces ranged between 240 N at 1W in spring and 100 N at 4W and 5W in summer ( Figure 9A.). Also, mean frictional resistance forces generally increased with subsequent scrapings of the same sample, *i.e.* with decreasing levels of biofouling ( Figure 9B.). Significant relationships between the frictional resistance data and the biofouling parameters *Richness, TBiom, BIOM, TDens,* and *DENS* were found (RELATE Rho between 0.24 and 0.40) ( Table 4A.). In addition, the frictional resistance data and the biofouling parameters *Richness, TBiom, TDens,* and *Thickness* were negatively correlated (Pearson correlations between -0.40 and -0.72) ( Table 4B.).

## Discussion

In this study, as could be expected for this region, variations in seawater parameters and biofouling characteristics, such as composition, richness, and abundance (measured as biomass and density), were observed across the three surveyed seasons: spring, summer, and autumn. Although some succession in biofouling colonization was observed in each season, the presence of hard-fouling organisms such as barnacles after only one week of submersion is aligned with a more ‘probabilistic model’ of colonization ( Clare *et al.,* 1992; Maki & Mitchell, 2002) rather than a ‘successional model’. Among the early colonisers is a non-indigenous species (NIS), the Australasian barnacle *A. modestus*, which was found with great frequency and density after one to three weeks of samples submersion. It should be highlighted that colonisation of artificial structures by NIS offshore doesn’t necessarily occur after such a short period. For example, in coastal areas such as the study site, colonisation can be enhanced by the many artificial substrates provided by ports and harbours. On the other hand, offshore conditions can be more challenging for larvae due to lower food availability and the need for larvae to travel greater distances to find suitable settlement sites.

It is well documented that higher temperatures typically found in spring and summer favour the reproductive and growth rates of marine organisms ( *e.g.* Gili & Petraitis, 2009; Newell & Branch, 1980). In this study, the highest biofouling biomass and abundance were registered in autumn, associated with longer submersion periods (six and eight weeks of submersion) that were not evaluated in spring and summer (maximum four and five weeks of submersion, respectively). Given longer submersion times, biofouling growth would most probably be more substantial in spring and summer. Therefore, in temperate to cold regions we recommend scheduling biofouling-related maintenance activities after the warmer seasons, namely in summer, to minimize the number of inspection and biofouling removal activities until the next season ( *e.g.* the next spring) most suitable for the breeding, spawning, and settlement of numerous biofoulers ( *e.g.* Anil *et al.,* 2012; Hellio & Yebra, 2009; Kupriyanova *et al.,* 2001). Additionally, summer generally offers longer and more frequent good weather windows, allowing for operators to conduct maintenance activities with lower costs and risks.

Biofouling biomass and thickness, both of which are mostly associated with sessile macrofoulers ( *e.g.* macroalgae, bryozoans, barnacles, mussels, calcareous tubeworms), are critical biofouling parameters affecting several marine industries ( *e.g.* Jusoh & Wolfram, 1996; Miller & Macleod, 2016; Tiron *et al.,* 2012; Titah-Benbouzid & Benbouzid, 2017; Yang *et al.,* 2017). In the present study, biofouling biomass and thickness were quite lower than those registered in more hydrodynamic locations offshore ( *e.g.* European Biofouling Database; Vinagre *et al.,* 2020) and at a nearby harbour after a similarly short submersion period ( Vinagre, 2023). This suggests that biofouling growing for short periods under sheltered conditions would have minimal impact on the loading, drag, or surface diameter of structures/components, compared to more hydrodynamic sites. It must be noted that biofouling biomass and thickness measurements taken out of water can be representative of underwater conditions but may not accurately reflect it. For example, organisms such as algae, tunicates and mussels incorporate water and have natural buoyancy which will reduce their effective weight underwater. These organisms, along with others such as arborescent bryozoans, can also exhibit increased volume underwater, thus representing greater thickness than that observed in dry conditions.

Besides contributing largely to biofouling weight and thickness, sessile macrofoulers can also cause physical damage to structures/components, for example damaging the substrates or their protective coatings by boring into them, when pulled by currents and waves, or during removal activities. Consequently, detrimental issues may arise concerning different types of corrosion ( *e.g.* Blackwood *et al.,* 2017; Jia *et al.,* 2019; Kleemann, 1996; Videla & Herrera, 2005). In the present study, corrosion was observed in the experimental setup after one week of submersion in components untreated against marine-induced corrosion (for example, on stainless steel nuts and washers used to tighten the caps) as well as in sections of NC samples (possibly owed to inefficient waterproofing of the untreated portion by the end caps) after four to five weeks of submersion in summer. This reinforces the importance of employing adequate anti-corrosion techniques on metallic substrates used in marine conditions even if for short periods. Cathodic protection has been extensively used on steel structures, and new corrosion monitoring techniques have been demonstrated and deployed. Recent anti-corrosion techniques proposed include applying thermally sprayed aluminium which has proven capability to protect steel substrates ( *e.g.* Syrek-Gerstenkorn *et al.,* 2019; Syrek-Gerstenkorn *et al.,* 2020; Vinagre *et al.*, 2022) or laser-cladded materials which in this study showed good anti-corrosive efficiency.

The results of the frictional resistance tests suggest that during these early colonization stages the slippery nature of biofouling could be acting as a ‘lubricant’ leading to a general trend of increasing forces being generated with decreasing biofouling levels. If early colonization stages can be accepted as a safe time interval to perform biofouling-related inspection and maintenance activities, then physical control, for example grooming, water jetting/cavitation or acoustic methods ( *e.g.* Legg *et al.,* 2015), could be an option to maintain the components’ integrity and equipment functionality and performance. However, because biofouling composition and growth are very variable and influenced by many factors ( *e.g.* Hellio & Yebra, 2009; Vinagre *et al.,* 2020), the definition of ‘acceptable biofouling growth’ will be project-specific, for example depending on the type of structure/component and its functional requirements ( *e.g.* free-moving versus static), the site location ( *e.g.* latitude, seawater temperature, distance to shore) and hydrodynamic conditions ( *e.g.* current velocity and wave exposure), and the bathymetry ( *e.g.* shallow versus deep waters) and depth ( *e.g.* surface versus mid water column) at which the structure/component is positioned.

The definition of “acceptable biofouling growth” may also be dependent on legislation ( *e.g.* EU Directive 2008/56/EC, EU Regulation 1143/2014) that aims to prevent or manage the introduction and spread of NIS. NIS may pose serious ecological threats by competing with, predating on, and/or excluding indigenous organisms, affecting community composition and structure, and potentially causing habitat modifications ( *e.g.* Cook *et al.,* 2014; Crooks, 2002; Lengyel *et al.,* 2009), consequently affecting ecosystems functioning and ecosystem services provision. Because MRE equipment can act as stepping stones facilitating the dispersion of NIS between artificial and natural habitats ( *e.g.* Adams *et al.*, 2014; De Mesel *et al.*, 2015), such effects can occur far away from the MRE deployment site. Also, it is very much possible that the dispersion of NIS is further enhanced if the organisms that reproduce sexually ( *e.g.* barnacles, mussels) or propagules of organisms that reproduce asexually through budding or fragmentation ( *e.g.* bryozoans, hydrozoans) are not recovered after using physical control strategies such as scraping or grooming.

Thus, it is valuable for the preservation of marine ecosystems and for MRE project developers to implement biosecurity risk management plans that can appropriately address biofouling and NIS propagation on their equipment at sea ( *e.g.* Cook *et al.,* 2014; Payne *et al.,* 2014), for example, through the development and installation of biofouling monitoring techniques. This should be especially considered for MRE projects undertaken in areas where numerous NIS are registered, such as those next to shipping lanes, commercial harbours, or nearshore/offshore, for example in the North Sea ( *e.g.* De Mesel *et al.,* 2015; Kerckhof *et al.,* 2018; Vinagre *et al.,* 2020). An important outcome to the developers could be that such management plans support or complement environmental impact assessments, potentially increasing the acceptability of projects and speeding up the licensing process.

## Conclusions

The findings of this study underscore the significance of managing biofouling during the early stages of colonisation. By mitigating the early attachment of biofoulers, there is a potential to curtail and delay subsequent biofouling, not only enhancing the preservation of materials and prolonging the interval between necessary biofouling-related maintenance operations but also reducing the possibility of NIS settlement and propagation, which can have profound ecological impacts.

The recommendation to schedule biofouling-related maintenance activities post-peak growth and reproduction periods, typically observed in warmer seasons within temperate to cold environments, emerges as a practical strategy. Adopting such a temporal approach could lead to a reduction in the frequency of cleaning operations, particularly before the subsequent growing season conducive to the breeding, spawning, and settlement of various key biofouling organisms.

It is necessary to acknowledge the limitations of this study, namely the very different environmental conditions of the study site compared to offshore and the fact that seasonal variations could not be assessed in different years. While providing valuable insights into early biofouling occurrences, the experimental design may not fully capture the dynamics of biofouling development. Therefore, it is crucial to interpret the present findings with caution. In light of these considerations, this research serves as a foundation, unveiling the need for more comprehensive investigations to refine our understanding of biofouling patterns and their implications for material preservation. Future studies should be conducted in offshore environments, with extended duration of experimental tests and larger sample sizes to ensure robust and representative results. In doing so, knowledge of biofouling dynamics can be advanced and preventive measures for the sustainable operation of MRE projects optimised.

## Data Availability

Zenodo: Experimental insights on biofouling growth in marine renewable structures, http://www.doi.org/10.5281/zenodo.6974716 ( Vinagre *et al*., 2024a) This project contains the following underlying data: Open Research Europe_Biological data.xlsx (Biofouling data) Zenodo: Experimental insights on biofouling growth in marine renewable structures, http://www.doi.org/10.5281/zenodo.10966268 ( Vinagre *et al*., 2024b) This project contains the following underlying data: Open Research Europe_Friction forces_all samples.xlsx Open Research Europe_Friction forces_subsequential scrapings.xlsx Zenodo: Experimental insights on biofouling growth in marine renewable structures, http://www.doi.org/10.5281/zenodo.10966299 ( Vinagre *et al*., 2024c) Data are available under the terms of the Creative Commons Attribution 4.0 International license (CC-BY 4.0) Underlying data.
