# Supplementary material for: Experimental insights on biofouling growth in marine renewable structures
Source: Open Res Eur. 2024 Jun 19;2:108. Originally published 2022 Sep 5. [Version 2] doi: 10.12688/openreseurope.14854.2 (PMC11329864; doi:10.12688/openreseurope.14854.2)
Supplement: Peer review history

# Reviewer report: Reviewer response for version 2

**DOI:** 10.21956/openreseurope.19083.r41545

**Published:** 2024-08-16

**Authors:** Andrew J Guerin

**Affiliations:** 1 Independent Researcher, Toronto, Canada

The article is substantially improved from the previous version. Additional details on the methodologies and analyses, along with a much deeper exploration of the data make this paper much more informative than it was. I thank the authors for the work that has gone into this revision. I have no additional comments at this stage; it is now acceptable for publication.

Is the study design appropriate and does the work have academic merit?

Partly

Is the work clearly and accurately presented and does it cite the current literature?

Partly

If applicable, is the statistical analysis and its interpretation appropriate?

Partly

Are all the source data underlying the results available to ensure full reproducibility?

Yes

Are the conclusions drawn adequately supported by the results?

Partly

Are sufficient details of methods and analysis provided to allow replication by others?

Partly

Reviewer Expertise:

Marine biofouling, aquatic ecology, non-native marine and freshwater organisms

I confirm that I have read this submission and believe that I have an appropriate level of expertise to confirm that it is of an acceptable scientific standard.

# Reviewer report: Reviewer response for version 2

**DOI:** 10.21956/openreseurope.19083.r41546

**Published:** 2024-06-27

**Authors:** Lenaïg Hemery

**Affiliations:** 1 Pacific Northwest National Laboratory, Washington, USA

The revised manuscript by Vinagre et al. is a substantial improvement from its earlier version a year ago. With all the changes made, I approve this new version, with the minor caveat that two relevant papers have been published since that the authors should consider discussing in theirs: Portas et al. and Want et al. Ref [1,2]

Is the study design appropriate and does the work have academic merit?

Partly

Is the work clearly and accurately presented and does it cite the current literature?

Yes

If applicable, is the statistical analysis and its interpretation appropriate?

Partly

Are all the source data underlying the results available to ensure full reproducibility?

Yes

Are the conclusions drawn adequately supported by the results?

Partly

Are sufficient details of methods and analysis provided to allow replication by others?

Partly

Reviewer Expertise:

Benthic ecology, marine renewable energy

I confirm that I have read this submission and believe that I have an appropriate level of expertise to confirm that it is of an acceptable scientific standard.

## References

[1] : Impact of hydrodynamics on community structure and metabolic production of marine biofouling formed in a highly energetic estuary. *Mar Environ Res* .2023;192: 10.1016/j.marenvres.2023.106241 106241 10.1016/j.marenvres.2023.106241 37922705

[2] 10.3390/jmse11112168

# Reviewer report: Reviewer response for version 1

**DOI:** 10.21956/openreseurope.16047.r32426

**Published:** 2023-07-28

**Authors:** Lenaïg Hemery

**Affiliations:** 1 Pacific Northwest National Laboratory, Washington, USA

The article by Vinagre & Fonseca is interesting and describes results from a field study relevant to the development of the marine renewable energy industry. However, the manuscript would benefit from several edits to the text to clarify some parts, and from additional data analyses to identify any correlation between biofouling and frictional resistance. See comments in the PDF file provided here.

Is the study design appropriate and does the work have academic merit?

Partly

Is the work clearly and accurately presented and does it cite the current literature?

Yes

If applicable, is the statistical analysis and its interpretation appropriate?

Partly

Are all the source data underlying the results available to ensure full reproducibility?

Yes

Are the conclusions drawn adequately supported by the results?

Partly

Are sufficient details of methods and analysis provided to allow replication by others?

Partly

Reviewer Expertise:

Benthic ecology, marine renewable energy

I confirm that I have read this submission and believe that I have an appropriate level of expertise to confirm that it is of an acceptable scientific standard, however I have significant reservations, as outlined above.

# Response

**Published:** 2024-04-12

**Authors:** Pedro Vinagre

**Affiliations:** WavEC Offshore Renewables, Portugal

**The authors would like to kindly thank the Reviewer for the time spent reading and reviewing our research paper. The extensive comments provided were extremely constructive and the article has been fully reviewed according to the feedback provided below.**  

 - Page 4 – “What were your controls? Did you have any samples without treatment?”

**The study didn’t include controls, i.e., samples without treatment. The authors recognise the importance of having controls vs. treatments in any study. However, the research conducted in the project aimed to infer the implications of biofouling growth in MRE structures that are treated against corrosion to be used in ocean environments. Thus, the project only tested samples that were treated against corrosion.**

 - Page 5 – “This is unnecessary information, you can delete.”

**While the authors understand that this information can be considered unnecessary for the scope of the present research, its inclusion was demanded by the Editorial team of the journal.**

 - Page 6 – “You said earlier that NC samples were omitted! Did you then redo the analyses without these samples?”

**Data was discarded only when corrosion was found in the NC samples . The data from the first deployments, when corrosion was not present, were used for analysis. This was further clarified earlier in the paper: “The NC1 and NC2 cylinders showed signs of corrosion during trials in September 2019, possibly owed to inefficient waterproofing of the untreated portion by the end caps. Corrosion can influence biofouling composition and growth, for example increasing the substrates roughness and physical-chemical properties. Therefore, data of biofouling growing in those samples in September 2019 were discarded from the analyses to avoid biased results and the NC samples were not used for further deployments (see deployment strategy below)”.**

 - Page 7 – “Any data analysis you could do to correlate the frictional resistance forces with BIOM, DENS, TBiom, or TDens? As is, these results leave a taste of "so what", try to dig a little deeper if you can.”

**The RELATE routine and Pearson correlations were used to analyse relations/correlations between frictional resistance forces and biofouling parameters (Richness, TBiom, BIOM, TDens, DENS and Thickness) and among the biofouling parameters.**

 - Page 11 – “Don't you think that the biofouling control measures that you suggest earlier would help disperse NIS? Could you list other measures that would not and be good for developers to implement?”

**We agree. It was added earlier in that section “Also, it is very much possible that the dispersion of NIS is further enhanced if the organisms that reproduce sexually (e.g. barnacles, mussels) or propagules of organisms that reproduce asexually through budding or fragmentation (e.g. bryozoans, hydrozoans) are not recovered after using physical control strategies such as scraping or grooming.” Other biofouling control measures were not listed because the feasibility of using them will depend on many factors such as the specific MRE technology and the type of equipment (e.g. static vs free moving), deployment location, environmental conditions, and regulatory requirements.** **The authors would like to kindly thank, once again, for the thoroughly made revision which has represented an asset for our research. We will be available for any questions/suggestions/corrections that you might raise.** **Best regards,** **The Authors**

# Reviewer report: Reviewer response for version 1

**DOI:** 10.21956/openreseurope.16047.r32422

**Published:** 2023-07-13

**Authors:** Andrew J Guerin

**Affiliations:** 1 Independent Researcher, Toronto, Canada

Vinagre and Fonseca study the early stages of the colonisation of surfaces in a site in Portugal, in the context of the development of a renewable energy device component. They record several biofouling metrics and assess community composition.

**Overall issues:**

 1) It is not completely clear what the purpose of this study was, and no hypotheses are stated; “to increase understanding on biofouling community structure” is very vague. Since the study was part of a larger project developing a power take-off system for renewable energy devices, it was apparently designed around testing components for this device. The data generated were undoubtedly important for the device developers, but it is not clear what the study contributes to our broader understanding. The measurement of frictional resistance during scraping is relatively novel, but the authors do not really explain why this data is useful or interesting. Given the small sample sizes, and the fact that link between the test samples and operational devices is weak, the strength of the evidence supporting the recommendations is limited.

 2) Since all data were collected in one year, ‘season’ is not replicated. It is therefore not strictly valid to claim that seasonal effects have been assessed. Biofouling assemblages change over time, and in the location studied, seasonal changes are very likely to be important drivers of the observed differences. However, multiple years data are needed to distinguish seasonal differences from those resulting from other short- and long-term changes. The authors should be more careful in their language when discussing these temporal effects.

 3) Some aspects of the data have not been fully analysed. There are some technical details of the data analysis and presentation that need to be clarified (see below), and some places where more detail is required.

 4) It is always good to see the data being made available, but there is little metadata to help the user understand what is actually provided. 

**Details:**

Introduction:

 a) “Marine biofouling is a natural process”. Especially since this is not a specialist journal, it is important to define what ‘marine biofouling’ is, here at the start of the paper.

 b) The text on climate change is a bit tenuous. Not all research has to be tied to climate change to be of value. The current text is also a little confusing. It is mentioned that climate change may be detrimental to some organisms that often make up fouling communities, but that sounds like it would be a good thing! However, marine biofouling is often extensive and rapidly-developing in warmer regions, so an important point that is missed here is that biofouling in temperate and polar seas could become more severe with ocean warming.

 c) To say that mechanical techniques are ‘capable of being totally efficient’ is not really correct. True, in optimal conditions, correctly applied, on a flat surface, mechanical techniques could remove most fouling. However, it seems unlikely that they would be able to remove everything, including all larvae and microfouling. Their negative sides are not mentioned. They will not be effective on moving parts, complex surfaces and niche areas (seawater intakes, vessel sea chests, heat exchangers, etc.) which are often problem areas for biofouling and are common on renewable energy devices. Mechanical techniques could also damage/remove existing anti-fouling and anti-corrosive coatings.

 d) It is true that deployment/maintenance of equipment could be timed to minimise fouling, but is this really realistic? Are deployment/maintenance schedules flexible enough? Operators may select summer for maintenance because this is when ocean conditions (wind, wave) are best for offshore operations and therefore lower-risk.

Methods:

 e) More information is needed on the measurement of friction forces. How many measurements for each sample and/or at what sampling frequency were they collected? There are a lot of numbers for each sample at each sampling time in the extended data, it would be useful to know why there are differences in the amount of data for each sampling event.

 f) Why was PERMANOVA used for analysis of univariate measures? I do not have a problem with this method but no reason is given for why a conventional ANOVA (on raw or suitably-transformed data) was not acceptable. Were the data very heteroscedastic, for example?

 g) For biofouling thickness, how many measurements were taken on each sample at each sampling event - just one, or are the data an average of several measurements? This should be made clear.

 h) Why were the data for some univariate variables (TBiom, TDens) square-root transformed for analysis? This is often done to help data meet ANOVA assumptions, but since PERMANOVA was used, such transforms may not be necessary. For the multivariate analyses (BIOM, DENS) why was the same (square-root) transform used? The justification for transforming multivariate data is usually to prevent one or two abundant species from dominating the analysis, so it seems odd (at first glance) that the same transform was used as for the univariate variables. Why were SIMPER analyses then run on untransformed data? It would seem more logical to use the same transforms as for the PERMANOVA. Whatever the reasons for the transforms that you used, it is important to justify these decisions.

 i) Once the authors decided to combine the LC/NC data, why were no PERMANOVA analyses carried out on the multivariate data (BIOM, DENS)? This is very important to demonstrate the significance of any differences among ‘seasons’ and deployment durations. The SIMPER analyses are useful for examining patterns of resemblances and identifying important species, but they do not show whether or not these differences are statistically significant in the first place.

Results:

 j) It seems that the differences among seasons in DO were not statistically significant. Therefore it is not appropriate to say that DO was greater in spring. Similarly in Table 2A the DO value for spring should not be highlighted as being greater if the difference was not significant.

 k) Table 2 - generally only statistically significant differences should be highlighted. Then ‘Significantly greater ( *p* < 0.05) numbers are presented in bold’ can be stated in the legend. Also, since the legend stated that the values are mean ± standard deviation, it is not necessary to repeat this with an * and a footnote under the table.

 l) There is very little information on the fouling composition and the actual differences among samples taken at different times and after different lengths of deployment. This is particularly notable since the primary stated aim of the work was to increase understanding of community structure. The data are available for download, but something should be presented in the paper, even if just to highlight which were the key taxa identified by SIMPER. A plot giving the proportional contributions of different taxonomic groups (eg. % biomass for algae, crustacea, etc.) at different times/sampling durations would be more informative.

 m) More description of the multivariate patterns would be worthwhile. The PCO plots are useful but some discussion of these would be useful.

 n) What does it mean to say that the SIMPER agrees with the overall trends in TBiom/TDens? There should be more detail about the actual patterns.

 o) The big problem with the friction data is that there does not appear to have been any statistical analysis, which limits what we can infer from the data.

 p) More information is needed to make sense of Figure 5, particularly sample sizes.

 q) Does 5A show the mean ± sd of all 6 (or 8) samples? Are these differences statistically significant?

 r) For 5B, why these 3 samples from these three time periods? Are they generally representative of other samples at other times? Some information is needed on why these were selected. I assume that for each bar, the mean and SD are those of all the measurements collected during the scraping of each sample. Again, are any of these differences statistically significant?

 s) 5B it is not all clear what is meant by subsequent scrapings. There is nothing about subsequent scrapings in the methods. I have to admit that I find this confusing.

Discussion:

 t) It is important to note that the weight added to a structure by biofouling is not the same as the fresh weight of those organisms in air. The density of the organisms is important - soft fouling species such as algae, tunicates, etc. will incorporate a lot of water and will have some natural buoyancy which will reduce their weight in the water. In these cases it is the increased roughness and hydrodynamic drag resulting from fouling by these species which increases structural loading.

 u) Similarly, the actual thickness of biofouling in water will differ from what is measured using calipers at the surface.

 v) The discussion seems to link frictional resistance/structural loading in water with the frictional resistance measurements obtained during scraping. It is very unlikely that frictional resistance during scraping is a good proxy for hydrodynamic load in operational conditions, and no evidence is provided (or cited) that the force required to remove fouling organisms is correlated with loading on fouled structures in this way. If such evidence exists, it should be mentioned here. If the authors were not intending to make this implied link, they should be clearer, and separate the discussion regarding scraping from that regarding structural loading.

 w) “In fact, with regards to frictional resistance, it was found that during these early colonization stages the slippery nature of biofouling could be acting as a ‘lubricant’ leading to lower forces generated from scraping the samples in areas with biofouling compared to areas without biofouling.” It is not clear where this inference has come from. If it is from the results of this study, the authors need to be clearer about how they arrived at this conclusion, and what data support it. If it has come from literature, additional citations would be needed to support this claim.  

**Minor/Typographical:**

Abstract:

- Instead of “small-scale wave energy components” perhaps ‘small-scale wave energy device components’ or something similar?
- “forces generated to scrape” - something like ‘frictional resistance forces generated during scraping’ (as used elsewhere in the paper) would be better.

Introduction:

- I am not familiar with standard terms in the MIC literature, but is it really correct to say that MIC is ‘initiated’ by micro-organisms, or is it just accelerated/enhanced?
- Another concern related to biofouling is that it creates opportunity for NIS…” perhaps the meaning would be clearer if this read: ‘Another concern related to biofouling of renewable energy structures is that…”.

Results:

- “several crustaceans fauna amphipods were observed”. I’m not sure what this means.

Discussion:

- “…NIS using the biofouling…” Perhaps ‘NIS within the biofouling assemblages’ would be preferable, since NIS are part of the biofouling assemblages, not something separate.

Is the study design appropriate and does the work have academic merit?

Partly

Is the work clearly and accurately presented and does it cite the current literature?

Partly

If applicable, is the statistical analysis and its interpretation appropriate?

Partly

Are all the source data underlying the results available to ensure full reproducibility?

Yes

Are the conclusions drawn adequately supported by the results?

Partly

Are sufficient details of methods and analysis provided to allow replication by others?

Partly

Reviewer Expertise:

Marine biofouling, aquatic ecology, non-native marine and freshwater organisms

I confirm that I have read this submission and believe that I have an appropriate level of expertise to state that I do not consider it to be of an acceptable scientific standard, for reasons outlined above.

# Response

**Published:** 2024-04-12

**Authors:** Pedro Vinagre

**Affiliations:** WavEC Offshore Renewables, Portugal

**The authors would like to kindly thank the Reviewer for the time spent reading and reviewing our research paper. The extensive comments provided were extremely constructive and the article has been fully reviewed according to the feedback provided below.**

 1) It is not completely clear what the purpose of this study was, and no hypotheses are stated; “to increase understanding on biofouling community structure” is very vague. Since the study was part of a larger project developing a power take-off system for renewable energy devices, it was apparently designed around testing components for this device. The data generated were undoubtedly important for the device developers, but it is not clear what the study contributes to our broader understanding. The measurement of frictional resistance during scraping is relatively novel, but the authors do not really explain why this data is useful or interesting. Given the small sample sizes, and the fact that link between the test samples and operational devices is weak, the strength of the evidence supporting the recommendations is limited.

**The original aim of this research is to understand and preliminarily evaluate the forces generated from removing different biofouling organisms in the samples (e.g. barnacles vs. algae). However, it was difficult to relate the forces measured with the specific occurrence of organisms in the samples. Nonetheless, the study provided interesting results:**

1. **It allowed to characterise the biofouling communities growing during increasing periods in three different seasons.**
2. **It highlights the importance of using adequate biofouling management and anticorrosive strategies.**

 
**Following your comments, we have:** **(i) updated the title of the paper;** **(ii) included several modifications throughout the paper to make its scope and impact better perceived by the reader;** **(iii) included a final comment on the conclusions section detailing the further work that is needed for a more comprehensive understanding of biofouling in metallic structures.**

 2) Since all data were collected in one year, ‘season’ is not replicated. It is therefore not strictly valid to claim that seasonal effects have been assessed. Biofouling assemblages change over time, and in the location studied, seasonal changes are very likely to be important drivers of the observed differences. However, multiple years data are needed to distinguish seasonal differences from those resulting from other short- and long-term changes. The authors should be more careful in their language when discussing these temporal effects. **The authors agree. Although the biofouling communities were characterised during three different seasons, no replication across years was possible. Thus, the discussion on temporal effects now takes these considerations into account.**

 3) Some aspects of the data have not been fully analysed. There are some technical details of the data analysis and presentation that need to be clarified (see below), and some places where more detail is required. **All the comments below have been fully addressed.**

 4) It is always good to see the data being made available, but there is little metadata to help the user understand what is actually provided. **Following your comment, some of the data previously provided as *Extended data* has now been incorporated into the paper.**

 Details:

 Introduction:

 a) “Marine biofouling is a natural process”. Especially since this is not a specialist journal, it is important to define what ‘marine biofouling’ is, here at the start of the paper. **A description of biofouling was added in the first sentence of the introduction.**

 b) The text on climate change is a bit tenuous. Not all research has to be tied to climate change to be of value. The current text is also a little confusing. It is mentioned that climate change may be detrimental to some organisms that often make up fouling communities, but that sounds like it would be a good thing! However, marine biofouling is often extensive and rapidly-developing in warmer regions, so an important point that is missed here is that biofouling in temperate and polar seas could become more severe with ocean warming. **These aspects were incorporated in the text.**

 c) To say that mechanical techniques are ‘capable of being totally efficient’ is not really correct. True, in optimal conditions, correctly applied, on a flat surface, mechanical techniques could remove most fouling. However, it seems unlikely that they would be able to remove everything, including all larvae and microfouling. Their negative sides are not mentioned. They will not be effective on moving parts, complex surfaces and niche areas (seawater intakes, vessel sea chests, heat exchangers, etc.) which are often problem areas for biofouling and are common on renewable energy devices. Mechanical techniques could also damage/remove existing anti-fouling and anti-corrosive coatings. **The sentence was rephrased accordingly.**

 d) It is true that deployment/maintenance of equipment could be timed to minimise fouling, but is this really realistic? Are deployment/maintenance schedules flexible enough? Operators may select summer for maintenance because this is when ocean conditions (wind, wave) are best for offshore operations and therefore lower-risk. **These aspects are now mentioned in the Discussion section.**

 Methods:

 e) More information is needed on the measurement of friction forces. How many measurements for each sample and/or at what sampling frequency were they collected? There are a lot of numbers for each sample at each sampling time in the extended data, it would be useful to know why there are differences in the amount of data for each sampling event. **For each cylinder, it was done one scraping event (except for the cylinders scraped more than once, as mentioned in the text) along the area available for colonisation (presented in the text). During each scraping, frictional resistance forces were acquired at a 50 Hz frequency (this has been included in the paper). The number of measurements along a cylinder depended on the velocity of scraping, i.e., faster scraping resulted in fewer measurements. Unfortunately, this was a first version of the scraping system, and we didn’t have a way to measure the velocity of scrapings (this has been added to the text in the Methods section). Additionally, differences in the number of measurements may also have resulted from removing outliers from the data (e.g., associated with the acceleration at the start or deceleration at the stop of the scraping event owed to the tightness of the plastic scraper to the samples) (this has also been added to the text).**

 f) Why was PERMANOVA used for analysis of univariate measures? I do not have a problem with this method but no reason is given for why a conventional ANOVA (on raw or suitably-transformed data) was not acceptable. Were the data very heteroscedastic, for example?

**PERMANOVA was used for different reasons, for example (i) it is a non-parametric method that can be used both for multivariate and univariate data; and (ii) being based on permutation tests, PERMANOVA is especially useful when sample sizes are small or there are unequal groups sizes, which is the case of this specific research** **. A comment on this has been included in the paper.**

 g) For biofouling thickness, how many measurements were taken on each sample at each sampling event - just one, or are the data an average of several measurements? This should be made clear. **Only one sample was taken as the highest point (associated with barnacles, bryozoans, or other organisms) from the cylinders surface. This has been clarified in the text.**

 h) Why were the data for some univariate variables (TBiom, TDens) square-root transformed for analysis? This is often done to help data meet ANOVA assumptions, but since PERMANOVA was used, such transforms may not be necessary. For the multivariate analyses (BIOM, DENS) why was the same (square-root) transform used? The justification for transforming multivariate data is usually to prevent one or two abundant species from dominating the analysis, so it seems odd (at first glance) that the same transform was used as for the univariate variables. Why were SIMPER analyses then run on untransformed data? It would seem more logical to use the same transforms as for the PERMANOVA. Whatever the reasons for the transforms that you used, it is important to justify these decisions. **The square-root transformation was done to reduce the influence of naturally abundant species (such as barnacles) in the analyses. This dominance was observed both for biomass and abundance and was observed using either the total biofouling data (univariate data, TBiom and TDens) or the individual taxa data (multivariate data, BIOM and DENS). This explanation was added to the text. The SIMPER analysis was repeated using the same transformation (square-root), and the methods and results sections are now reflecting these changes.**

 i) Once the authors decided to combine the LC/NC data, why were no PERMANOVA analyses carried out on the multivariate data (BIOM, DENS)? This is very important to demonstrate the significance of any differences among ‘seasons’ and deployment durations. The SIMPER analyses are useful for examining patterns of resemblances and identifying important species, but they do not show whether or not these differences are statistically significant in the first place. **Following your suggestion, PERMANOVA analyses based on BIOM and DENS have now been conducted and their results have been added to the paper.**

 Results:

 j) It seems that the differences among seasons in DO were not statistically significant. Therefore it is not appropriate to say that DO was greater in spring. Similarly in Table 2A the DO value for spring should not be highlighted as being greater if the difference was not significant. **While the authors agree with the reviewer, we highlight that the aim was not to present the statistical differences but to show how the values changed among seasons. The text and the title of Table 2 were changed to reflect this accordingly.**

 k) Table 2 - generally only statistically significant differences should be highlighted. Then ‘Significantly greater (p < 0.05) numbers are presented in bold’ can be stated in the legend. Also, since the legend stated that the values are mean ± standard deviation, it is not necessary to repeat this with an * and a footnote under the table. **As mentioned for the previous comment, in Table 2 we meant to highlight changes among seasons and increasing submersion periods, and not the statistical differences. Hence, the higher values were kept in bold. The text “mean ± standard deviation” was removed from the Table 2 title according to your suggestion.**

 l) There is very little information on the fouling composition and the actual differences among samples taken at different times and after different lengths of deployment. This is particularly notable since the primary stated aim of the work was to increase understanding of community structure. The data are available for download, but something should be presented in the paper, even if just to highlight which were the key taxa identified by SIMPER. A plot giving the proportional contributions of different taxonomic groups (eg. % biomass for algae, crustacea, etc.) at different times/sampling durations would be more informative. **The section on community structure and PERMANOVA and SIMPER results was restructured to better highlight patterns among seasons and submersion periods. SIMPER data was previously made available as *Extended data*. Part of that data have been included in the paper as Table 5.**

 m) More description of the multivariate patterns would be worthwhile. The PCO plots are useful but some discussion of these would be useful. **Multivariate patterns are now better described in the Results section and discussed in the Discussion section.**

 n) What does it mean to say that the SIMPER agrees with the overall trends in TBiom/TDens? There should be more detail about the actual patterns. **As mentioned earlier, and following your suggestions, the section on SIMPER results was restructured to better highlight patterns among seasons and submersion periods. Also, part of the SIMPER data which was previously made available as *Extended data* is now included in the paper as Table 5.**

 o) The big problem with the friction data is that there does not appear to have been any statistical analysis, which limits what we can infer from the data. **Following your suggestion, PERMANOVA analyses based on the frictional resistance forces (univariate data) were conducted and their results were added to the paper.**

 p) More information is needed to make sense of Figure 5, particularly sample sizes. **The mean values and standard deviation were calculated using all the samples within a submersion period within a season. For example, the mean + standard deviation of the sample “summer 1W” was calculated using the frictional resistance measurements of the three “1W” samples of “summer”. A clarification on this has been added to the text in the Methods section.**

   q) Does 5A show the mean ± sd of all 6 (or 8) samples? Are these differences statistically significant?

**As mentioned for the previous comment, the mean + sd was calculated using all the samples within a submersion period within a season. PERMANOVA analyses based on the frictional resistance forces (univariate data) were conducted and their results were added to the paper.**

 r) For 5B, why these 3 samples from these three time periods? Are they generally representative of other samples at other times? Some information is needed on why these were selected. I assume that for each bar, the mean and SD are those of all the measurements collected during the scraping of each sample. Again, are any of these differences statistically significant? **As it was now clarified in the Methods section, some of the cylinders were scraped more than once until complete cleaning was achieved** **to measure the friction forces generated from scraping decreasing levels of biofouling (macrofouling – biofilm – no biofouling). This was done only for some summer and autumn samples which stayed in water for longer submersion periods (4-8W). The previous version of the paper presented only some examples, and now it presents all the samples which were scraped more than once. Those samples are now identified in Table 1. PERMANOVA analyses were now conducted, and their results were added to the paper.**

 s) 5B it is not all clear what is meant by subsequent scrapings. There is nothing about subsequent scrapings in the methods. I have to admit that I find this confusing. **The reason for the subsequent scraping of some cylinders was presented for the previous comment. This information was added to the text in the Methods section.**

 Discussion:

 t) It is important to note that the weight added to a structure by biofouling is not the same as the fresh weight of those organisms in air. The density of the organisms is important - soft fouling species such as algae, tunicates, etc. will incorporate a lot of water and will have some natural buoyancy which will reduce their weight in the water. In these cases it is the increased roughness and hydrodynamic drag resulting from fouling by these species which increases structural loading. **The authors agree, and that notion was incorporated into the discussion. The authors would like to note that “density” refers to the number of individuals per square meter and not the mass per unit of volume.**

 u) Similarly, the actual thickness of biofouling in water will differ from what is measured using calipers at the surface. **The authors agree. This is especially true when the thickness is related mostly to, for example, soft fouling like algae and ascidians, or arborescent bryozoans. This was incorporated into the discussion.**

 v) The discussion seems to link frictional resistance/structural loading in water with the frictional resistance measurements obtained during scraping. It is very unlikely that frictional resistance during scraping is a good proxy for hydrodynamic load in operational conditions, and no evidence is provided (or cited) that the force required to remove fouling organisms is correlated with loading on fouled structures in this way. If such evidence exists, it should be mentioned here. If the authors were not intending to make this implied link, they should be clearer, and separate the discussion regarding scraping from that regarding structural loading. **The intention was not to relate frictional resistance/scraping data with hydrodynamic load, and the authors agree that such data is not a good proxy for hydrodynamic load in operational conditions.** **This was clarified in the discussion. Also, differences between measurements underwater and outside of water are now highlighted.**

 w) “In fact, with regards to frictional resistance, it was found that during these early colonization stages the slippery nature of biofouling could be acting as a ‘lubricant’ leading to lower forces generated from scraping the samples in areas with biofouling compared to areas without biofouling.” It is not clear where this inference has come from. If it is from the results of this study, the authors need to be clearer about how they arrived at this conclusion, and what data support it. If it has come from literature, additional citations would be needed to support this claim. **The inference comes from the results presented in the paper, as shown in Figures 9A and 9B (previously Figures 5A and 5B) and mentioned in the text. This has been clarified in the text.**

Minor/Typographical:

Abstract: Instead of “small-scale wave energy components” perhaps ‘small-scale wave energy device components’ or something similar? **The sentence in the abstract was changed accordingly.**

****“forces generated to scrape” - something like ‘frictional resistance forces generated during scraping’ (as used elsewhere in the paper) would be better. **The sentence in the abstract was changed accordingly.**

****

**Introduction:** I am not familiar with standard terms in the MIC literature, but is it really correct to say that MIC is ‘initiated’ by micro-organisms, or is it just accelerated/enhanced? **Yes, according to the literature (for example the references used in the paper) biofouling (namely microfouling) can initiate MIC.**

****Another concern related to biofouling is that it creates opportunity for NIS…” perhaps the meaning would be clearer if this read: ‘Another concern related to biofouling of renewable energy structures is that…”. **The process occurs in many different sectors. The sentence was changed to “Another concern related to biofouling of MRE structures (and others installed at sea)”.**

 Results: “several crustaceans fauna amphipods were observed”. I’m not sure what this means. **It has been corrected.**

 Discussion: “…NIS using the biofouling…” Perhaps ‘NIS within the biofouling assemblages’ would be preferable, since NIS are part of the biofouling assemblages, not something separate. **The sentence was changed accordingly.** **The authors would like to kindly thank, once again, for the thoroughly made revision which has represented an asset for our research. We will be available for any questions/suggestions/corrections that you shall raise.** **Best regards,** **The Authors**
